# A self-attention-based deep learning model for identifying key genes in insect pupal metamorphosis

**DOI:** 10.1186/s12864-026-13128-5

**Published:** 2026-07-06

**Authors:** Fangrong Liu, Yong Cao, Songping Qian, Xingyu Tong, Junhui Liu, Yan Zhang, Jiaying Zhu, Jiawei Mao, Xingke Yang, Jiasheng Hao, Youjie Zhao

**Affiliations:** 1 College of Big Data and Intelligent Engineering, Southwest Forestry University, Kunming, China; 2 College of Mathematics and Physics, Southwest Forestry University, Kunming, China; 3 College of Forestry, Southwest Forestry University, Kunming, China; 4 Institute of Zoology, Chinese Academy of Sciences, Beijing, China; 5 College of Life Sciences, Anhui Normal University, Wuhu, China

**Keywords:** Insect proteome, Metamorphosis, Key genes, Natural language processing, Deep learning, Attention mechanism

## Abstract

Metamorphosis is the major innovation in the evolution of insect, playing an important role in environmental adaptation and biodiversity formation. However, it has been a challenge to identify the key genes in insect pupal metamorphosis. In this study, we constructed a library of word embeddings based on Word2vec for 293 insect protein sequences from 14 orders. A gene network classification model (GNCM) based on deep learning (DL) and self-attention mechanisms (SAM) was designed to identify key genes by calculating their importance (weights) in the metamorphosis of insect pupae. Empirical studies demonstrated that GNCM achieved a significantly better performance than other algorithms, including ANN, SVM, XGBoost, BiGRU, and BiLSTM, classification accuracy and interpretability. The results showed that GNCM identified 1,048 high-weight gene families, and differential expression analysis revealed that high-weight genes exhibited significantly higher expression levels than low-weight genes during the pupal stage. KEGG annotation showed that these genes were involved in functions with developmental, apoptosis, and immunity, which are crucial for insect metamorphosis. This study not only develops a novel artificial intelligence approach applicable to the identification of key genes, but also provided new insights for understanding the development of insect metamorphosis.

**Supplementary Information:**

The online version contains supplementary material available at https://doi.org/10.1186/s12864-026-13128-5.

## Introduction

Insects originated during the Early Ordovician period (~ 479 million years ago) [1, 2] and evolved through a developmental process from wingless to winged and from hemimetabolous to holometabolous [3, 4]. These organisms comprise more than 50% of all known animals [5], with more than 98% of them undergoing metamorphosis during their life cycle [6]. The formation of metamorphosis promotes insect prosperity and diversity [7, 8], allowing them to utilize different habitats and resources in order to adapt to changing environments. Hemimetabolous insects develop directly into adults from the final larval (nymph) stage, whereas holometabolous insects molt into the pupal stage and undergo a remarkable process of morphological reorganization [9, 10]. The mechanisms underlying the formation of pupae in holometabolous insects are a key issue in evolutionary biology. However, the molecular mechanisms underlying the successful evolution of insect metamorphosis have not yet been systematically and comprehensively investigated. In particular, the molecular regulatory mechanisms governing the metamorphosis process during the pupal stage of holometabolous insects remain a mystery.

In previous studies, insect metamorphosis was shown to involve the co-regulation and expression of numerous genes, as well as the complete remodeling of cells, tissues and organs (holometabolous). For example, Prothoracicotropic hormone (PTTH) initiates the onset of molting and metamorphosis [11], Ecdysone is converted to 20-hydroxyecdysone (20E), which initiates and regulates the process of moulting and metamorphosis [12], and juvenile hormone (JH) regulates the direction of metamorphosis [13, 14]. And the transcription factor Krüppel-homolog 1 (Kr-h1) regulates pupal development by inhibiting the transition from larva to adult in hemimetabolous insects and pupal development in holometabolous insects [15]. In addition, the transcription factor chronologically inappropriate morphogenesis (chinmo), which is also essential for maintaining the larval stage of insects, together with *broad-complex*(*br*) and Eip93F(Ecdysone-induced protein 93 F), constitutes a trinity of mutually repressive master genes of insect metamorphosis [16], which regulate metamorphic processes in insects. Among them, the *br* regulates the formation and development of hemimetabolous insect wings and holometabolous insect pupae [17, 18], whereas the Eip93F plays a central role in the formation of the adult stage [19]. Although these genes are crucial for elucidating their key roles in the metamorphosis process during the insect pupal stage, it remains unclear whether other genes are also involved in the molecular regulatory mechanisms. These studies based on traditional functional gene identification methods often require extensive biological experiments to validate their reliability, usually at a high cost. Therefore, how to use an efficient and low-cost artificial intelligence method to identify genes critical to insect pupal metamorphosis is the main problem to be addressed in this study.

Among existing machine learning (ML) methods, most models rely on traditional algorithms such as Support Vector Machines (SVM), Random Forests (RF) and Xtreme Gradient Boosting (XGBoost) for training [20–22]. This approach requires significant manual effort to select effective parameters from vast amounts of manually designed features for feature selection. While these methods are effective with small datasets, they often struggle to identify efficient training features when handling large-scale datasets. With the rapid development of artificial intelligence, deep learning (DL) [23] is a way that enables better and more efficient data mining and analysis. Compared to traditional ML [24, 25], DL is widely used in data mining and analysis tasks due to its ability to automatically extract features and higher accuracy [26]. In recent years, DL models have been widely used in tasks such as molecular structure prediction [27], enhancer prediction [28], and gene expression prediction [29] in the field of biology. For example, the DL model AlphaFold3 can predict the combined structure of complexes including proteins, nucleic acids, small molecules, ions, and modified residues, which is difficult to achieve with traditional ML algorithms [30]. However, the interpretability of DL during the inference stage remains limited. In the task of identifying key genes for insect metamorphosis, interpretability is equally important as model performance. Attention mechanisms [31, 32] can compute weights for the input gene sequences of deep learning models during training, thereby capturing the model’s focus on specific sequences. Applying AM to identify key genes during the pupal metamorphosis of insects still presents significant challenges.

In this study, using protein sequences from 293 insect species across hemimetabolous and holometabolous taxa, we designed a gene network classification model (GNCM), which is based on bidirectional gated recirculation unit (BiGRU) and self-attention mechanisms (SAM) with the goal of identifying genes that are critical for influencing the development of insect pupal metamorphosis. In addition, we also investigated the functional enrichment and differential expression of genes related to pupal metamorphosis. Our study shows the great potential of GNCM in identifying key genes for insect pupal metamorphosis and lays the foundation for a better understanding of the molecular mechanisms of insect metamorphosis.

## Materials and methods

### Taxon sampling

The proteomes of 293 insects were obtained from National Center for Biotechnology Information (NCBI) (https://www.ncbi.nlm.nih.gov/) database [33], with protein-coding genes as the raw data samples. According to the different types of metamorphosis, these protein sequences are divided into two classes: hemimetabolous and holometabolous. Among them, the hemimetabolous insects cover 8 orders with 36 species, and the holometabolous insects cover 6 orders with 257 species. The distribution is as follows (Table 1): Odonata (2), Ephemeroptera (2), Blattodea (3), Phasmatodea (1), Orthoptera (7), Phthiraptera (1), Thysanoptera (2), Hemiptera (18), Coleoptera (24), Lepidoptera (44), Siphonaptera (1), Neuroptera (1), Hymenoptera (83) and Diptera (104). Detailed information is given in Table S1.

**Table 1:** Protein sequences of 293 insects from 81 families in 14 orders

NO	Metamorphosis	Order	Family	Genus	Species	Total proteins
1	Hemimetabolous	Odonata	2	2	2	27,057
2	Hemimetabolous	Ephemeroptera	1	2	2	25,871
3	Hemimetabolous	Blattodea	3	3	3	42,160
4	Hemimetabolous	Phasmatodea	1	1	1	14,448
5	Hemimetabolous	Orthoptera	2	2	7	121,322
6	Hemimetabolous	Phthiraptera	1	1	1	10,758
7	Hemimetabolous	Thysanoptera	1	2	2	30,848
8	Hemimetabolous	Hemiptera	9	17	18	272,972
9	Holometabolous	Coleoptera	12	20	24	341,072
10	Holometabolous	Lepidoptera	13	29	44	600,114
11	Holometabolous	Siphonaptera	1	1	1	18,878
12	Holometabolous	Neuroptera	1	1	1	12,985
13	Holometabolous	Hymenoptera	19	55	83	956,107
14	Holometabolous	Diptera	15	36	104	1,475,724

### Workflow of GNCM

**Fig. 1:**
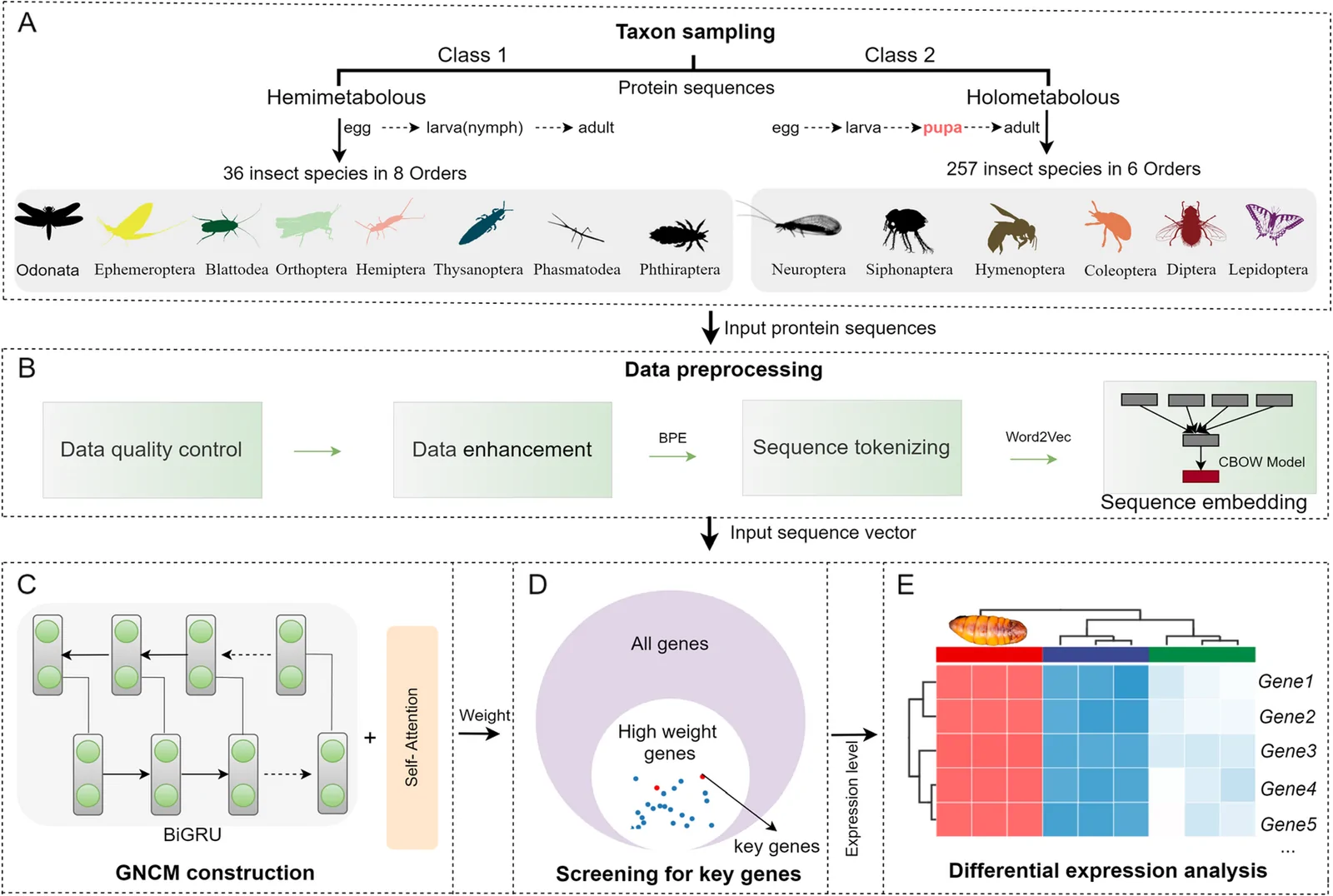
Workflow of GNCM to identify metamorphosis genes.** A** The protein sequences of 293 insect species, including 36 hemimetabolous and 257 holometabolous were selected as Class 1 and Class 2. **B** In the data preprocessing, the data quality control and data enhancement were carried out firstly, and the protein sequences were then subjected to the operations of sequence tokenizing and embedding, to generate vectorized representations. **C** The GNCM was constructed by combining BiGRU with the self-attention. **D** The output of the GNCM was used to screen the key genes related to pupal metamorphosis. **E** Differential expression analysis of highly weighted genes. Images of representative taxa in the 14 orders were obtained from PhyloPic (http://phylopic.org)

Hemimetabolous and holometabolous of 293 insect species were used as classification labels for the experimental samples. The hemimetabolous and holometabolous were modeled as a classification problem of protein sequences (Fig. 1A). Based on these protein sequences, externally we use NLP to construct word embedding libraries, and then add AM to the BiGRU classification network to calculate the weights of different genes in the classification process. The workflow of GNCM used in this study mainly includes four parts: Data preprocessing (Fig. 1B), GNCM construction(Fig. 1C), Screening for key genes(Fig. 1D), and Differential expression analysis(Fig. 1E). The details of each part are described in the following subsections.

### Data preprocessing

#### Data quality control

To ensure the completeness and reliability of the protein sequences of each insect, we systematically evaluated all samples using BUSCO v5 (Benchmarking Tool for Universal Single Copy Orthologous Genes, v5) [34, 35]. BUSCO outputs include four key metrics: Complete (C, including Single-copy, S, and Duplicated, D), Fragmented (F), and Missing (M). Among them, the C reflects the overall integrity of the sequence, while the proportion of M and F suggests potential sequence deletion. In this study, we selected the OrthoDB database insecta_odb10 as the reference lineage, which contains a collection of highly conserved single-copy orthologous genes within insects, and is suitable for quality control of protein sequences. During the analysis, we ran the protein mode of BUSCO v5 to evaluate the protein sequences of insects, and adopted the default parameters to ensure the comparability of the results among different datasets. Based on the analysis results, we screened out datasets with high deletion rates or large fragmentation ratios, and only retained the sequence samples with high BUSCO scores for subsequent model construction and analysis, so as to ensure the reliability and scientific validity of the research results.

#### Data enhancement

To meet the sample requirements for deep learning, we expanded the protein sample size for holometabolous and hemimetabolous groups through two rounds of data augmentation. (1) Due to the limited number of samples from hemimetabolous insects, which could lead to lower prediction accuracy for certain categories, we performed oversampling to achieve a relatively balanced sample size between the two groups. (2) We used Orthofinder to cluster the genomic sequences of 293 insects with orthologous genes, and subsequently expanded the sample size by simulating real data [36]. First, we calculated the coverage of each Orthogroup based on the absence of Orthogroups across different species and excluded Orthogroups with excessively high absence rates to retain a set of gene families with high representativeness. Second, during each sampling batch, we randomly selected a fixed number of groups from the filtered Orthogroup set. For each species sample, we extract the corresponding protein sequences from the selected Orthogroups and concatenate them in the order of the predefined delimiters to construct new composite sequence samples. Therefore, in this study, we train, validate, and test the performance of GNCM using samples generated by simulating real data. To minimize model accuracy bias caused by the sampling strategy, we applied the same data construction workflow and model parameters to all methods in the model comparison experiments.

#### Sequence tokenizing

**Fig. 2:**
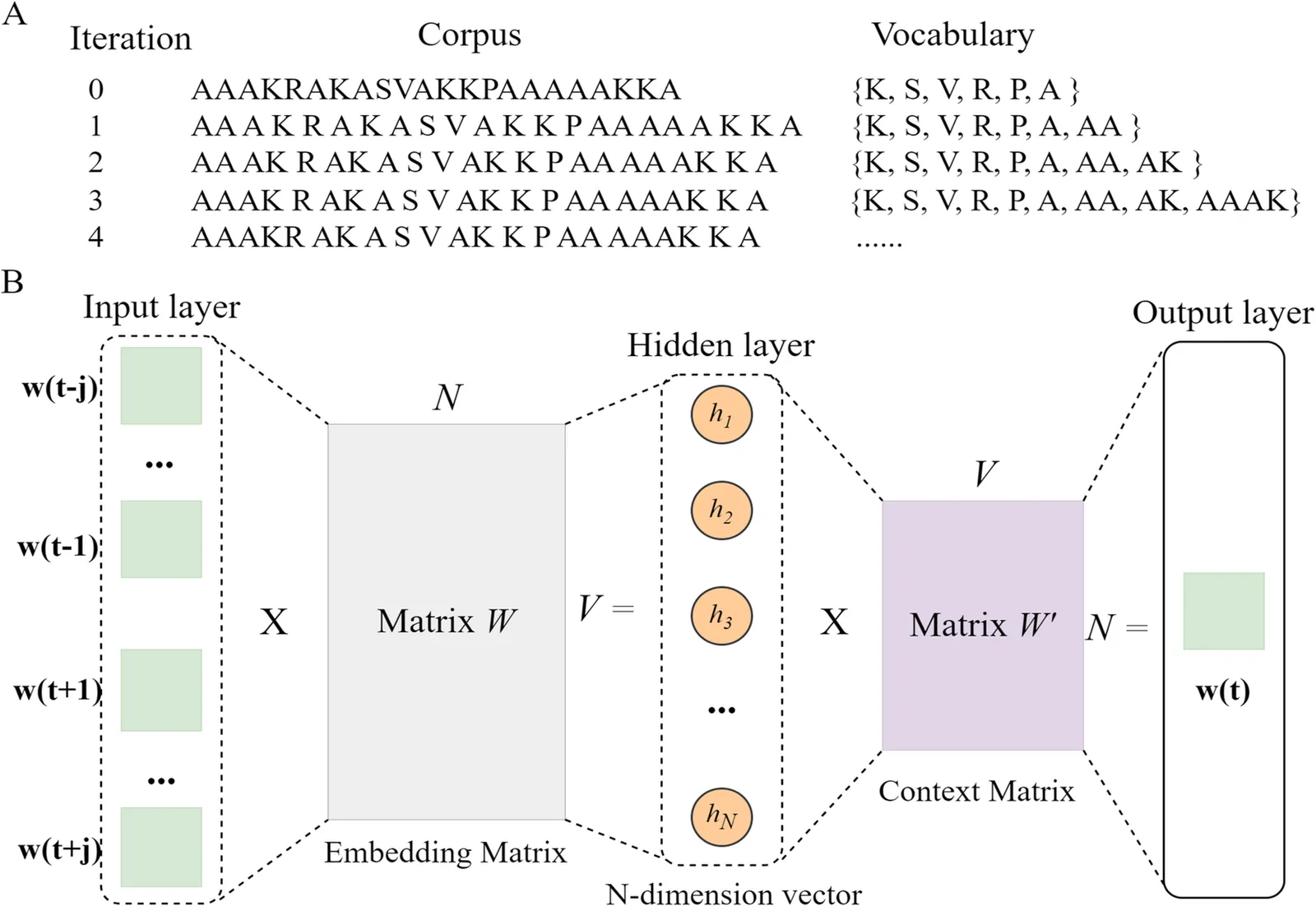
The process of BPE tokenization and word embedding model design in CBOW.** A** Represents the process of BPE to tokenize the protein sequences. It first initializes the vocabulary list, then counts the frequency of each subword pair, and finally generates a subword dictionary by merging the most frequent subwords to generate the subword dictionary. **B** The CBOW word embedding algorithm for Word2vec. It predicts the center word by context words. The context window of CBOW word embedding model in this study is set to 20, the embedding dimension is 150, and the number of training rounds is 5

BPE is a data compression algorithm that iteratively merges the most frequently occurring genome segments in a corpus to construct a fixed-size vocabulary list, which has been widely used in the field of NLP for word segmentation strategies [37, 38]. Based on the BPE algorithm, the protein sequences of 293 insects are processed for word segmentation, and Fig. 2A shows the detailed process of vocabulary construction in a given corpus. First, we initialize the vocabulary with all the unique characters in the corpus. Then, in each iteration, the character segment with the highest frequency of occurrence (e.g., AA in iteration 1 of Fig. 2A) is considered as a new word to be added to the vocabulary, and the corpus is updated by replacing all the same character segments with this new word. This iteration continues until the desired vocabulary size is reached.

In addition, common word segmentation methods include k-mer [39]. K-mer segmentation is essentially a rule-based approach that uses fixed windows to segment sequences in a systematic manner; however, it ignores potential long-range dependencies and complex semantic structures within the sequences, which may result in the loss of some valuable biological information. Therefore, this study builds upon the k-mer method to investigate the use of high-frequency k-mers (referred to as HFkmer) for protein sequence segmentation (Fig S1).

#### Sequence embedding

Word embedding is an important technique in the field of NLP, which overcomes the limitations of traditional one-hot coding methods by mapping words to a low-dimensional continuous vector space to capture semantic relationships. In this study, we use the CBOW algorithm in Word2vec [40] for word embedding training (Fig. 2B). The core idea behind the Skip-gram (Sg) algorithm is the opposite of that of CBOW: it predicts the probability of the target word based on the surrounding words (Fig S2). The amino acids of each protein sequence are replaced with their vector representations obtained from the pre-trained word embedding model, and then by taking the average of all the amino acid embedding vectors in that protein sequence as its feature representation.

In CBOW word embedding process, the context vector $\:{v}_{c}$ is obtained by summing and averaging the word vectors of the context words, which are denoted as $\:{\mathrm{w}}_{\mathrm{t}-\mathrm{j}}$,…,$\:{\mathrm{w}}_{\mathrm{t}-1}$,$\:{\mathrm{w}}_{\mathrm{t}+1}$,…,$\:{\mathrm{w}}_{\mathrm{t}+\mathrm{j}}$ with the window size of 2w, then the $\:{v}_{c}$ computation formula is as shown in Eq. (1).

1 $$v_c=\frac1{2w}\sum\limits_{-w\leq j\leq w,\neq0}\;v_{w_{t+j}}$$

where $\:{v}_{{w}_{t+j}}$ is the word vector of the word $\:{w}_{t+j}$ with j ranging from -c to c and j ≠ 0 (i.e., excluding the center word) when summed over all context words. The probabilistic prediction of the target word $\:{w}_{t}$ is computed by calculating the dot product of $\:{v}_{c}$ with the word vectors of all the words in the vocabulary as shown in Eq. (2).

2 $$\:P\left({w}_{t}\right|{w}_{t-j},\dots\:,{w}_{t-1},{w}_{t+1},\dots\:,{w}_{t+j})=\frac{exp({u}_{{w}_{t}}\cdot\:{v}_{c})}{\sum\:_{{w}^{{\prime\:}}\in\:\:V}exp({u}_{{w}^{{\prime\:}}}\cdot\:{v}_{c})}$$

where, $\:{\mathrm{w}}_{\mathrm{t}-\mathrm{j}}$,…,$\:{\:\mathrm{w}}_{\mathrm{t}-1}$,$\:{\mathrm{w}}_{\mathrm{t}+1}$,…,$\:{\mathrm{w}}_{\mathrm{t}+\mathrm{j}}$ denote the context words of the target word$\:\:{w}_{t}$, $\:{\mathrm{u}}_{{\mathrm{w}}_{\mathrm{t}}}$ denotes the word vectors of the target word$\:\:{w}_{t}$, $\:{\mathrm{u}}_{{\mathrm{w}}^{{\prime\:}}}$ denotes the vectors of all the words $\:{\mathrm{w}}^{{\prime\:}}$ in the vocabulary. The probability distribution is obtained by softmax function using negative log-likelihood loss function as shown in Eq. (3).

3 $$\:\mathcal{L}=-\sum\limits_{t=1}^{T}logP\left({w}_{t}\right|{w}_{t-j},\dots\:,{w}_{t-1},{w}_{t+1},\dots\:,{w}_{t+j})$$

where $\:\mathcal{L}$ denotes the loss function, and *T* is the total number of words in the corpus.

### GNCM construction

The construction of GNCM is mainly used to identify genes families that are crucial for insect metamorphosis. The GNCM model is developed based on PyTorch, and the Adam optimizer [41] is used for model training, with the learning rate set to 0.001. In general, an appropriate learning rate or combination of learning rates can accelerate model training and produce better or even optimal performance. If the learning rate is too small, it will take too long to reach the desired state; conversely, if the learning rate is too large, the algorithm may not converge. In addition, we use classification cross entropy as the loss function for model training. Because cross entropy is very effective in binary classification scenarios, it represents the difference between the true probability distribution and the predicted probability distribution. In addition, we set the number of epochs to 50 and the size of batch to 8 to train the GNCM model. We set dropout and early stopping strategies to avoid overfitting [42, 43]. Due to the differences in the length of the protein sequences, during the training process, we make the sequences of the same length by padding the end of the sequence with zeros (zeros represent any unknown amino acid type).

**Fig. 3:**
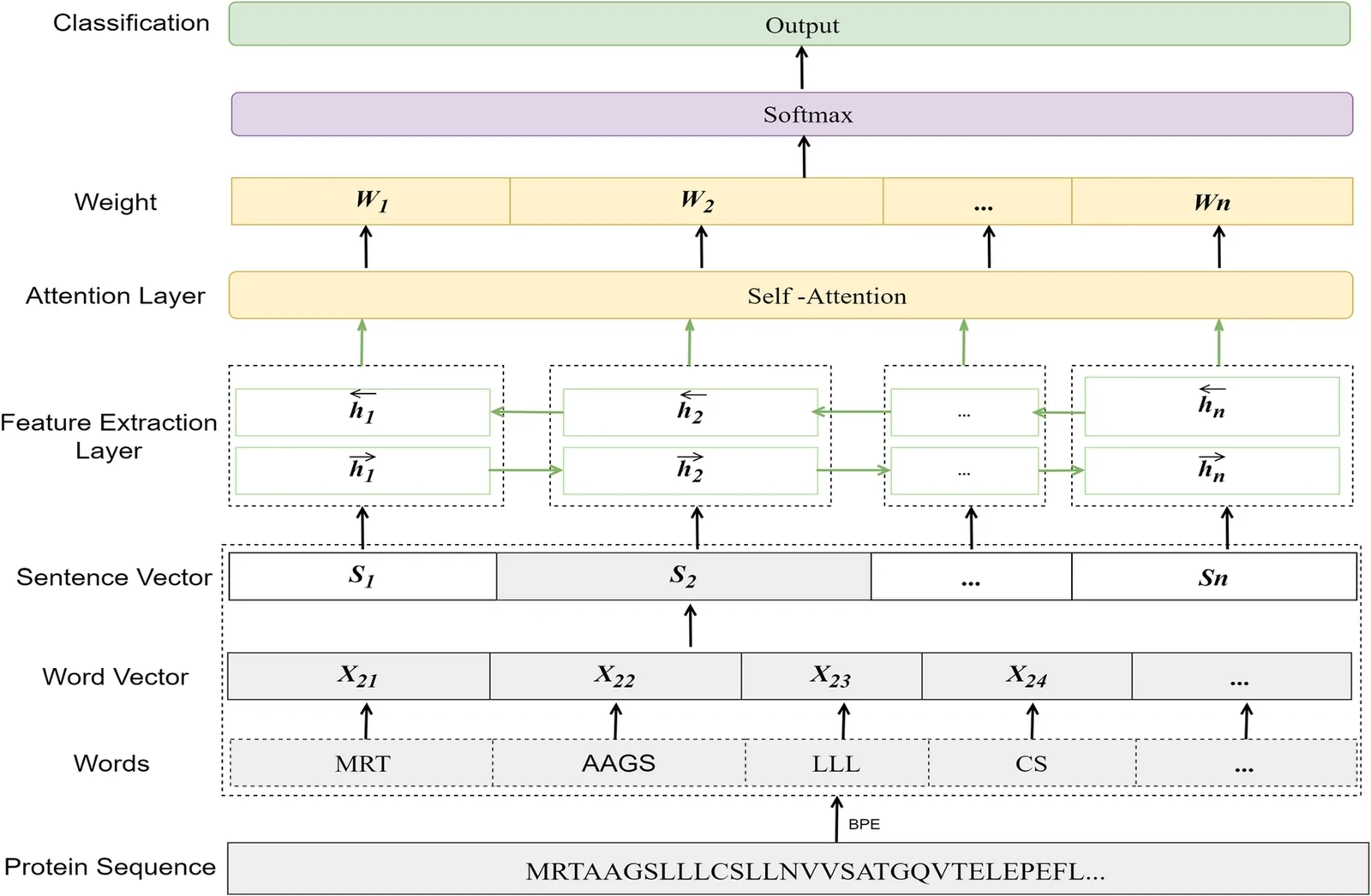
Architecture of GNCM. The input protein sequence is first tokenized into words using BPE. Each word is embedded as a vector $\:{X}_{ij}$. For each sentence, its representation $\:{S}_{i}$ obtained by averaging its constituent word vectors * .* These vectors are fed into the feature extraction layer, where a bidirectional recurrent network encodes contextual information and generates hidden representations $\:{\overrightarrow{h}}_{i},{\overleftarrow{h}}_{i}$. Self-attention layer is subsequently applied to capture long-range dependencies and assign different weights ($\:{W}_{i}$) to the features. Finally, the weighted feature representations are passed through a softmax classifier to produce the final output for protein sequence classification. Where $\:{X}_{i}$ and $\:{S}_{i}\:(i\:\in\:\:[\mathrm{1,2},\:...,\:n\left]\right)$ denote the input representation of the *ith* word and the *ith* sentence, respectively, and h denotes the hidden state

The architecture of GNCM used in our research is as follows (Fig. 3): first, the protein sequences of all species were preprocessed for normalization. Subsequently, each sequence is characterized by Word2Vec, and a 150-dimensional vector sequence is obtained as input. This input is then fed into BiGRU to capture bi-directional contextual dependencies, and the key information is weighted and aggregated by the AM. Finally, the gene family determination is realized through the fully connected layer and *softmax* activation to output the category probability.

The gated recurrent unit (GRU) uses a gating mechanism to monitor the state of a sequence without using a separate storage unit, which consists of an update gate $\:{\mathrm{z}}_{\mathrm{t}}$ and a reset gate $\:{\mathrm{r}}_{\mathrm{t}}$. Update gate $\:{\mathrm{z}}_{\mathrm{t}}$ controls how much information $\:{\mathrm{h}}_{\mathrm{t}}$ needs to keep from $\:{\mathrm{h}}_{\mathrm{t}-1}$ and how much information it needs to receive from $\:{\stackrel{\sim}{\mathrm{h}}}_{\mathrm{t}}$. Reset gate $\:{\mathrm{r}}_{\mathrm{t}}$ controls whether the computation of $\:{\stackrel{\sim}{\mathrm{h}}}_{\mathrm{t}}$ depends on $\:{\mathrm{h}}_{\mathrm{t}-1}$ or not. GRU can effectively alleviate the problem of long-distance dependence in LSTM and improve the computational efficiency, and it is calculated as follows:

4 $$\:{z}_{t}=\sigma\:\left({W}_{z}{x}_{t}+{U}_{z}{h}_{t-1}+{b}_{z}\right)$$

5 $$\:{r}_{t}=\sigma\:\left({W}_{r}{x}_{t}+{U}_{r}{h}_{t-1}+{b}_{r}\right)$$

6 $$\:{\stackrel{\sim}{\mathrm{h}}}_{\mathrm{t}}=tanh\left({W}_{h}{x}_{t}+{U}_{h}\left({r}_{t}\odot{h}_{t-1}\right)+{b}_{h}\right)$$

7 $$\:{h}_{t}=\:{z}_{t}\:\odot\stackrel{\sim}{{h}_{t}}+\left(1-{z}_{t}\right)\odot{h}_{t-1}$$

where $\:{\mathrm{h}}_{\mathrm{t}-1}$ and $\:{\mathrm{h}}_{\mathrm{t}}$ denote the hidden layer states at moments * t-1* and * t*, respectively. $\:{\stackrel{\sim}{\mathrm{h}}}_{\mathrm{t}}$ denotes the candidate states, * W* and * U* denote the weights, and b is the bias. σ is the Sigmoid function, and the output 0 is completely discarded, and 1 is completely retained.

We use BiGRU to process the sequence data, which consists of forward-propagating GRU units and backward-propagating GRU units. We unify the length of sequences by Padding technique so that the model can handle inputs of different lengths. BiGRU combines the forward and backward GRU to capture the bidirectional semantic information of sequences, which solves the problem of variable-length sequences, and also improves the computational efficiency and the performance of the model. The BiGRU is calculated as follows:

8 $$\:{\overrightarrow{h}}_{i}=\overrightarrow{\mathrm{G}\mathrm{R}\mathrm{U}}({\mathrm{s}}_{i},{\overrightarrow{\mathrm{h}}}_{\mathrm{i}-1})$$

9 $$\:{\overleftarrow{h}}_{i}=\overleftarrow{\mathrm{G}\mathrm{R}\mathrm{U}}({\mathrm{s}}_{\mathrm{i}},{\overleftarrow{\mathrm{h}}}_{\mathrm{i}+1})$$

10 $$\:{h}_{i}=[{\overrightarrow{h}}_{i},{\overleftarrow{h}}_{i}]$$

where $\:{\overrightarrow{h}}_{i}$ and $\:{\overleftarrow{h}}_{i}$ denote the forward and backward implicit variables of $\:{\overrightarrow{\mathrm{h}}}_{\mathrm{i}-1}$ and $\:{\overleftarrow{\mathrm{h}}}_{\mathrm{i}+1}$, respectively, for the ith sentence in the text. In this study, AM is added to BiGRU to dynamically adjust the weights of different parts of the input sequences, which enables the model to process the sequences more flexibly, and the attention scores are calculated as shown in Eq. (11).

11 $$\:{e}_{ij}=\frac{\left(Q{W}_{Q}\right){\left(K{W}_{K}\right)}^{T}}{\sqrt{{d}_{k}}}$$

where *Q* is the query matrix, *K* is the key matrix, $\:{W}_{Q}$ is the query weight moment, and $\:{W}_{K}$ the key weight matrix. $\:{d}_{k}$ is the dimension of the key vector, which is used for scaling dot product. Attention weight $\:{a}_{ij}$ is the result of score $\:{e}_{ij}$ after softmax function, which represents the attention weight of the *ith* query vector to the *jth* key vector. The attentional weight $\:{a}_{ij}$ is calculated as shown in Eq. (12) and finally the value vectors of the sentence are weighted and summed using the attentional weights as shown in Eq. (13).

12 $$\:{a}_{ij}=softmax\left({e}_{ij}\right)=\frac{exp\left({e}_{ij}\right)}{\sum\limits_{k=1}^{n}exp\left({e}_{ik}\right)}$$

13 $$\:{c}_{i}=\sum\limits_{j=1}^{n}{a}_{ij}{V}_{j}$$

where *n* denotes the length of the sequence and *V* is the value matrix, which represents the value representation of the input sequence after linear transformation. Each value vector $\:{V}_{j}$ corresponds to an element of the input sequence.

We used four performance evaluation metrics, Accuracy (𝐴𝑐𝑐), Precision (𝑃𝑟𝑒), Recall(𝑅𝑒), F1 score (𝐹1) and area under the receiver operating characteristic (ROC) curve (AUC) to assess the effectiveness of the GNCM in general. All metrics are positive, i.e., the larger the metrics the better the model, and it is calculated as follows:

14 $$\:\begin{array}{c}\:Acc=\frac{TP+TN}{TP+TN+FP+FN}\end{array}$$

15 $$\:\begin{array}{c}Pre=\frac{\mathrm{T}\mathrm{P}}{\mathrm{T}\mathrm{P}+\mathrm{F}\mathrm{P}}\end{array}$$

16 $$\:\begin{array}{c}Re=\frac{\mathrm{T}\mathrm{P}}{\mathrm{T}\mathrm{P}+\mathrm{F}\mathrm{N}}\end{array}$$

17 $$\:\begin{array}{c}\:F1=\frac{2\times\:\mathrm{P}\mathrm{r}\mathrm{e}\mathrm{c}\mathrm{i}\mathrm{s}\mathrm{i}\mathrm{o}\mathrm{n}\times\:\mathrm{R}\mathrm{e}\mathrm{c}\mathrm{a}\mathrm{l}\mathrm{l}}{\mathrm{P}\mathrm{r}\mathrm{e}\mathrm{c}\mathrm{i}\mathrm{s}\mathrm{i}\mathrm{o}\mathrm{n}+\mathrm{R}\mathrm{e}\mathrm{c}\mathrm{a}\mathrm{l}\mathrm{l}}\end{array}$$

where *TP* (True Positive) denotes the number of positive samples correctly classified, *TN* (True Negative) denotes the number of negative samples correctly classified, FP (False Positive) i.e., the number of misclassified as actual samples, and *FN* (False Negative) denotes the number of actual labels not predicted by the model in classification.

### Weight calculation for genes

In this study, the weights for gene families were derived from a real-world dataset consisting of 36 samples of hemimetabolous insects and 257 samples of holometabolous insects. The attention scores for each gene were aggregated to form the weight for the corresponding gene family. Through the attention weight calculation of the GNCM module (Fig.S3), we obtain the weight distribution$\:\:\left\{{\alpha\:}_{1},{\alpha\:}_{2},\dots\:,{\alpha\:}_{n}\right\}\:$for the query vector, where the weight value reflects the relevance of each key vector$\:\:{key}_{i}$ to the query vector. The value vector $\:{Value}_{i}$ corresponding to the high weight value $\:{\alpha\:}_{i}$ contributes more to the final attention value, while the value vector with a low weight value contributes less. The weights obtained by GNCM are arranged in descending order, and the top 5% Orthogroups with the highest weights are taken out as the high-weight genes identified by the classification model. Then, the key genes in this study are obtained by intersecting.

### Differential expression analysis

To retrieve transcriptome data of insects at different developmental stages, we searched NCBI’s Sequence Read Archive (SRA) Browser (https://www.ncbi.nlm.nih.gov/sra) using the species name and developmental stage as search terms to obtain RNA-seq SRA login numbers. Ultimately, we downloaded transcriptome data from 27 *Drosophila melanogaster* (*D. melanogaster*), and 24 *Spodoptera frugiperda* (*S. frugiperda*). Detailed information on the stage of transcriptome data for each species is provided in Table S2. For insect high-weighted genes identified by GNCM, we performed transcript assembly and abundance estimation using StringTie v2.2.1 [44]. Transcript abundance was normalized to transcripts per million (TPM) by calculating read coverage for each transcript, normalizing counts based on transcript length, and scaling based on total sequencing depth for each sample. Soft clustering analysis of genes was performed using Mfuzz software [45], processing standardized read counts based on the developmental process. We selected nine developmental stages. In addition, we used weighted gene co-expression network analysis (WGCNA) to perform module clustering of gene expression across different developmental stages [46].

## Results

### Data preprocessing results

**Fig. 4:**
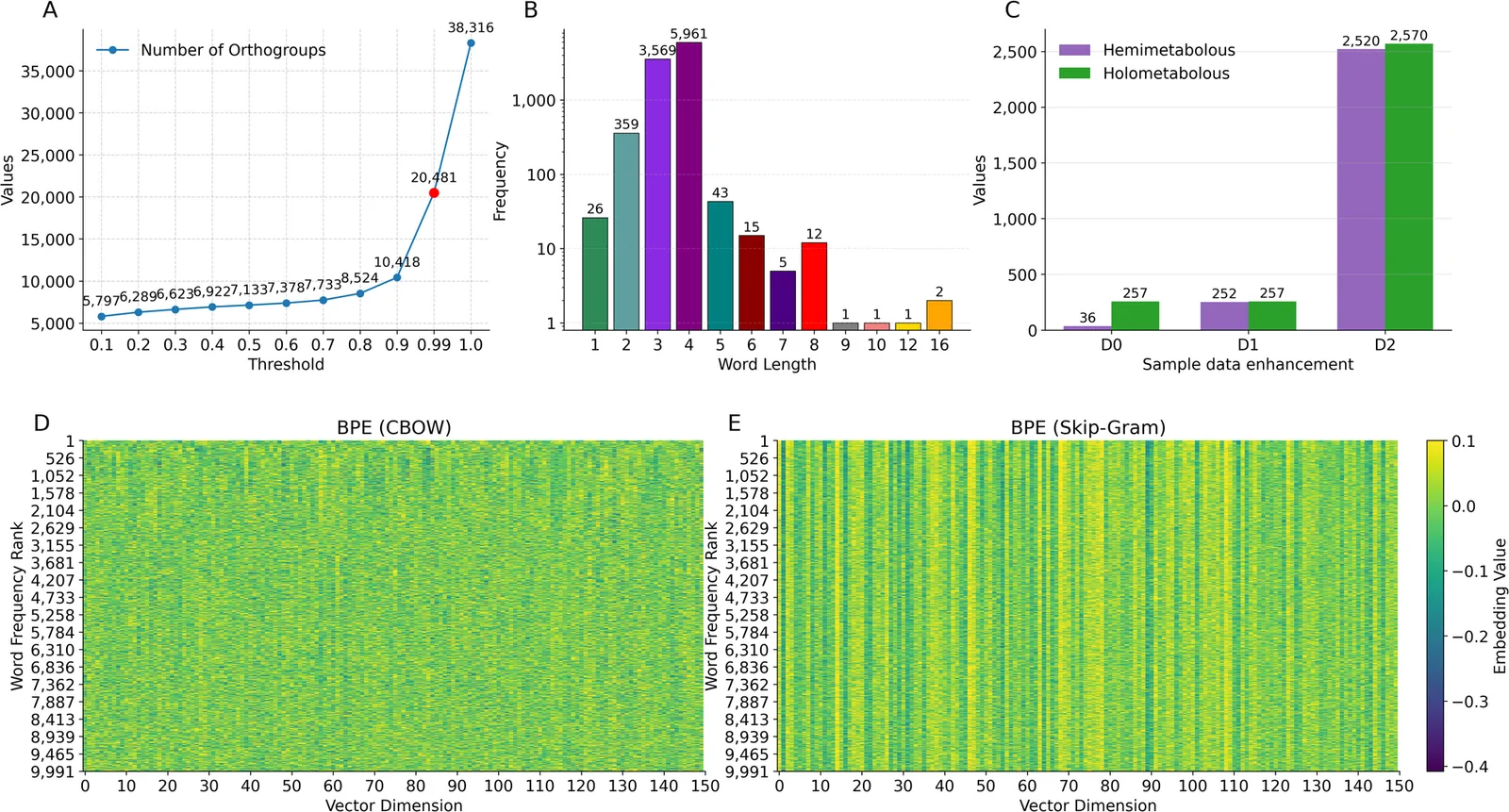
The results of data preprocessing.** A** Orthogroups’ length selection strategy, when the length is 20,481, it can cover 99% of species. A larger gap value means that there are fewer species with gene family in each Orthogroup. **B** BPE tokenizer frequency of words of different lengths. **C** Sample size after data enhancement, D0 represents the original sample, D1 is the number of samples after enhancement of hemimetabolous taxon samples, and D2 is the number of samples after gene family sampling and expansion. **D** Word2vec-based CBOW word embedding heatmap. **E** Word2vec-based Skip-gram word embedding heatmap. The horizontal axis represents the dimension of the embedding vector (from 1 to 150), the vertical axis represents the ranked frequency of words (from high to low), and the color shade reflects the magnitude and direction of the vector value in different dimensions. The yellow indicates a larger positive value, green indicates a larger negative value, and the darker the color, the more significant the activation of the embedding vector in that dimension

Protein sequences of 257 holometabolous and 36 hemimetabolous insects used to construct the GNCM were obtained from NCBI. Their phylogenetic relationships indicate that holometabolous insects evolved later than hemimetabolous insects (Fig.S4). The completeness assessment showed that the BUSCO values of these proteomes were all higher than 90%, with an average of 98.5% (Table S1). A total of 3,950,316 proteins with longest transcripts were obtained from these 293 insects, with the number ranged from 11,969 to 25,647 (Fig.S4, Table S1). Among them, the average gene number in hemimetabolous insects was about 15,151, while the value in holometabolous insects was 13,249. Based on the 3,950,316 protein sequences of 293 species, 38,316 Orthogroups were clustered using Orthofinder (Table S3). Subsequently, orthologous genes from 20,481 Orthogroups were selected to construct GNCM for simulated insect samples (Fig. 4A).

In order to meet the sample size requirements of deep learning, we simulated and expanded the samples. A total of 2,520 hemimetabolous and 2,570 holometabolous samples were obtained from 293 insects based on their gene family of 20,481 Orthogroups (Fig. 4B). The total 5,090 samples were divided into 3,562 training samples, 1,017 validation samples, and 511 test samples for GNCM in a ratio of 7:2:1. For each input sample, 13,000 sequences are extracted and encoded into a 150-dimensional embedding vector, which serves as the training input for the GNCM. All protein sequences of the 2,190 samples were vectorized by the 9,991 words and used to construct GNCM.

In this study, the parameters for the CBOW and Skip-gram word embedding methods were set identically: a window size of 20, an embedding dimension of 150, a minimum word frequency of 1,500, and 5 training iterations. The results show that the embedding heatmaps generated by the CBOW model generally exhibit more uniform and smooth features, with relatively continuous color transitions (Fig. 4D). Unlike the Skip-gram, no particularly prominent band-like clustering phenomena were observed across different dimensions (Fig. 4E). Therefore, during training, the CBOW tends to learn contextual information in a more globally averaged sense, resulting in a more stable distribution of features across dimensions and a better overall balance in the embedding space.

### Training and evaluation of GNCM

**Fig. 5:**
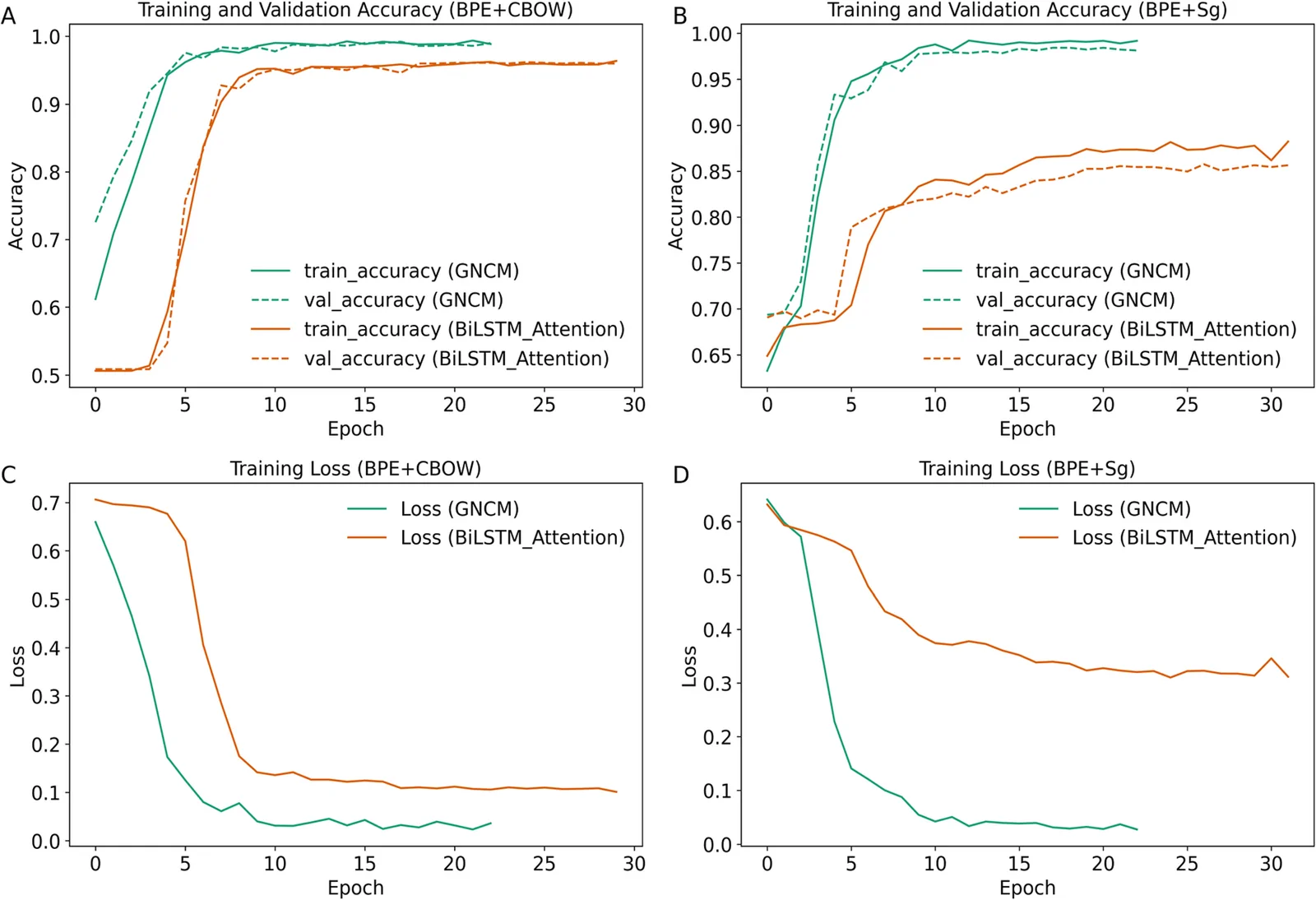
Comparison of training performance between BiGRU and BiLSTM with attention under different word embedding representations. **A** The training and validation accuracy of GNCM (BPE+CBOW). **B** The training and validation accuracy of GNCM (BPE + Sg). **C** The training and validation loss of GNCM (BPE+CBOW). **D** The training and validation loss of GNCM (BPE + Sg)

**Fig. 6:**
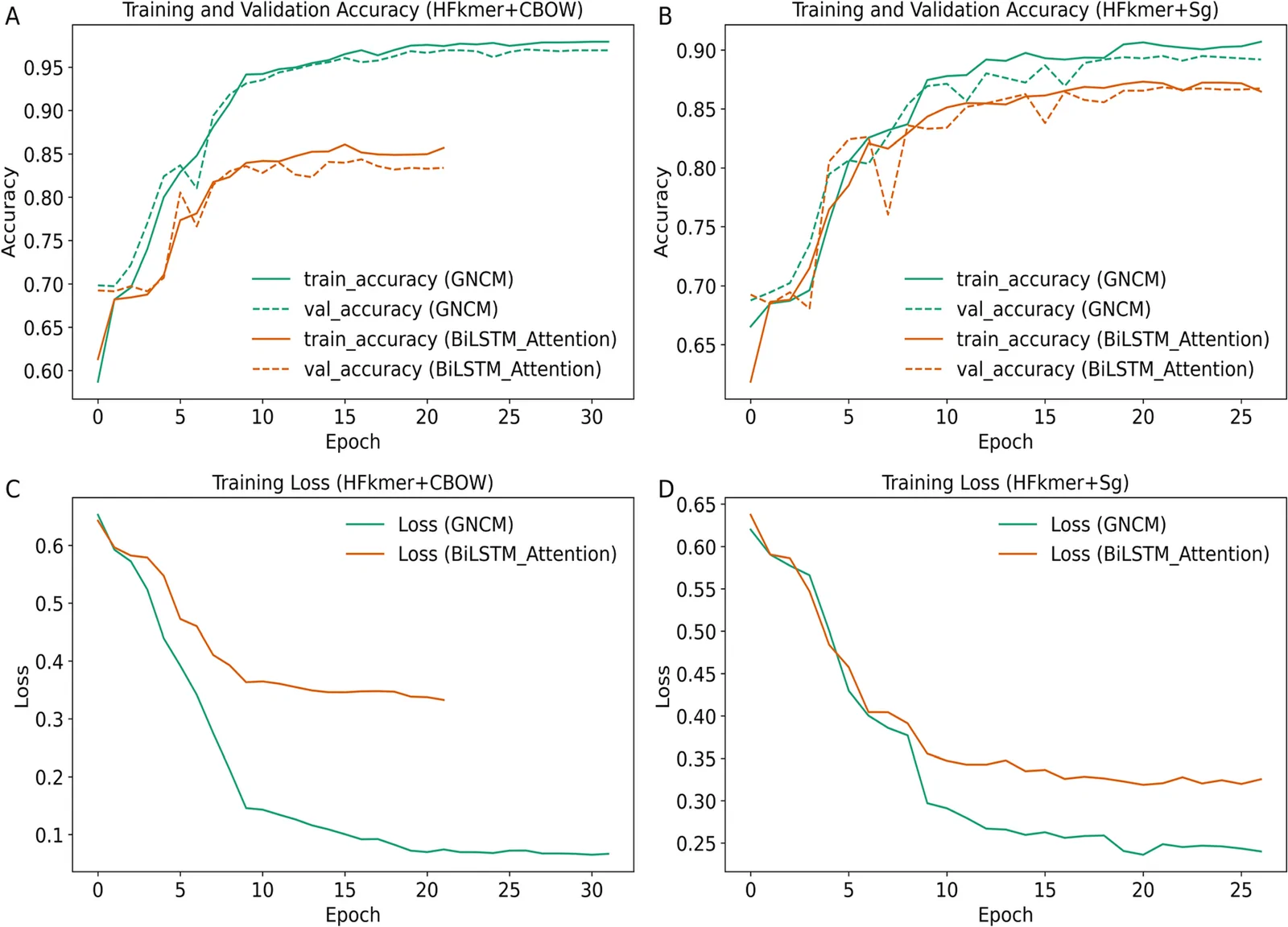
Comparison of training performance between HFkmer and BPE with attention under different word embedding representations.** A** The training and validation accuracy of GNCM (HFkmer+CBOW). **B** The training and validation accuracy of GNCM (HFkmer + Sg). **C** The training and validation loss of GNCM (HFkmer +CBOW). **D** The training and validation loss of GNCM (HFkmer + Sg)

It is important to note that during the training of deep learning models, close attention must be paid to potential overfitting issues. In this study, we not only employed “dropout” and “early stopping” strategies to prevent overfitting, but also continuously monitored the trends in model loss and accuracy using the training and validation sets. Figures 5A and C illustrate the trends in accuracy and loss during the training and validation phases of GNCM. After five training epochs, the loss and accuracy values remained largely stable. As training progressed, the attention layer learned high-level abstract features that were crucial for classification predictions, leading the model to converge to a state of high accuracy. In Fig. 5, we compare the training and validation accuracy and loss curves of GNCM based on BPE under the two word embedding methods (CBOW and Sg). The results show that the accuracy and loss of GNCM based on the BPE+CBOW and BPE + Sg combinations both outperform BiLSTM_Attention. Furthermore, Fig. 6 compares the accuracy and loss of GNCM using the HFkmer+CBOW and HFkmer + Sg combinations, which also outperform BiLSTM_Attention. It is worth noting that the BPE-based protein sequence feature construction method significantly outperforms HFkmer, while the CBOW-based protein sequence feature extraction significantly outperforms Sg (Table 2).

**Table 2:** A comparison of GNCM performance on the test set using different sequence tokenizing and word embedding methods

Sequence tokenizing	Word Embedding	Algorithm	Accuracy	F1	Precision	Recall	Cross-Entropy Loss
BPE	CBOW	GNCM	0.9960	0.9957	0.9947	0.9967	0.0126
	Sg	GNCM	0.9922	0.9922	0.9921	0.9923	0.0328
	CBOW	BiLSTM + SAM	0.9647	0.9647	0.9646	0.9648	0.1054
	Sg	BiLSTM + SAM	0.8686	0.8682	0.8781	0.8708	0.3380
HFkmer	CBOW	GNCM	0.9869	0.9869	0.9873	0.9865	0.0391
	Sg	GNCM	0.8072	0.8012	0.8257	0.7998	0.4263
	CBOW	BiLSTM + SAM	0.9935	0.9934	0.9934	0.9934	0.0140
	Sg	BiLSTM + SAM	0.8170	0.8107	0.8400	0.8090	0.4078

Here, we report the ablation test results for the GNCM framework. First, we compared the prediction performance across different embedding dimensions (Table 3). We set the embedding dimensions to 30, 50, 100, and 150 and compared their impact on model performance. Results indicate that GNCM achieves optimal performance and minimal loss at embedding dimension = 150, followed by 100, 50, and 30, while 30 yields the poorest results. Additionally, we conducted an ablation analysis on the deep learning framework within GNCM to investigate whether the attention layer enhances model predictive performance. Performance comparisons from ablation test revealed that this effect only became apparent after incorporating the attention layer.

**Table 3:** Results of the ablation analysis of the GNCM on test dataset

Algorithm	Accuracy	F1	Precision	Recall	Cross-Entropy Loss
GNCM with parameter embedding dim = 150	0.9960	0.9957	0.9947	0.9967	0.0126
GNCM with parameter embedding dim = 100	0.9757	0.9745	0.9700	0.9804	0.0328
GNCM with parameter embedding dim = 50	0.9676	0.9659	0.9627	0.9698	0.0752
GNCM with parameter embedding dim = 30	0.9474	0.9453	0.9393	0.9555	0.1173
BiGRU with parameter embedding dim = 150	0.7530	0.6998	0.7845	0.6919	0.5443

To validate the classification performance of GNCM, we first compared the classification performance of BiLSTM and BiGRU networks using BPE and HFkmer, respectively. The results showed that the classification accuracy of both models was below 0.76 (Table S7), which clearly demonstrates the contribution of SAM (Table 2). Second, we also compared the performance of GNCM, ANN, BiLSTM (bidirectional long short-term memory), SVM, and XGBoost (Table 4). Compared to the other four methods, GNCM demonstrates significant advantages across all five performance evaluation metrics. GNCM achieves the best classification results for all five performance evaluation metrics in each category, while methods such as SVM, XGBoost, and ANN perform poorly, with BiLSTM yielding the worst results. Overall, the findings indicate that GNCM excels in identifying insect pupal metamorphosis genes.

**Table 4:** Performance analysis results of the GNCM on the test dataset with other algorithms

Methods	Accuracy	F1	Precision	Recall	Cross-Entropy Loss
GNCM	0.9960	0.9957	0.9947	0.9967	0.0126
BiLSTM	0.7247	0.6720	0.7305	0.6670	0.5761
SVM	0.9555	0.9527	0.9536	0.9517	0.0954
ANN	0.9555	0.9516	0.9665	0.9415	0.3090
XGBoost	0.9312	0.9240	0.9500	0.9096	0.4913

### Weight acquisition and KEGG enrichment analysis

**Fig. 7:**
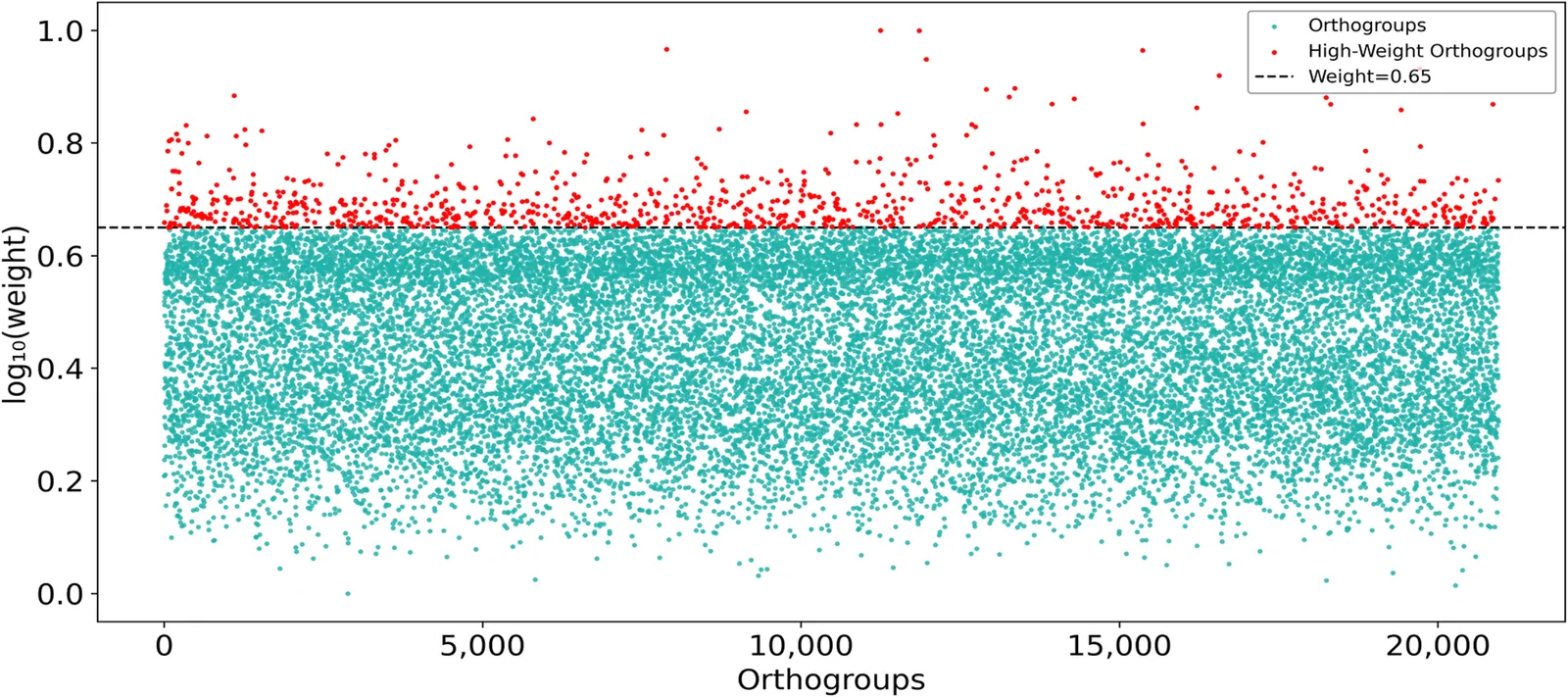
Weight distribution of gene families. High-weight Orthogroups accounts for 5%

The weights of Orthogroups were obtained from the AM of GNCM (Table S4). Among these Orthogroups, 1,048 Orthogroups (accounting for 5%) have weights exceeding 0.65 (Fig. 7). We designate the genes within these 1,048 orthogroups as high-weight genes, and subsequent analyses will be based on these genes.

**Fig. 8:**
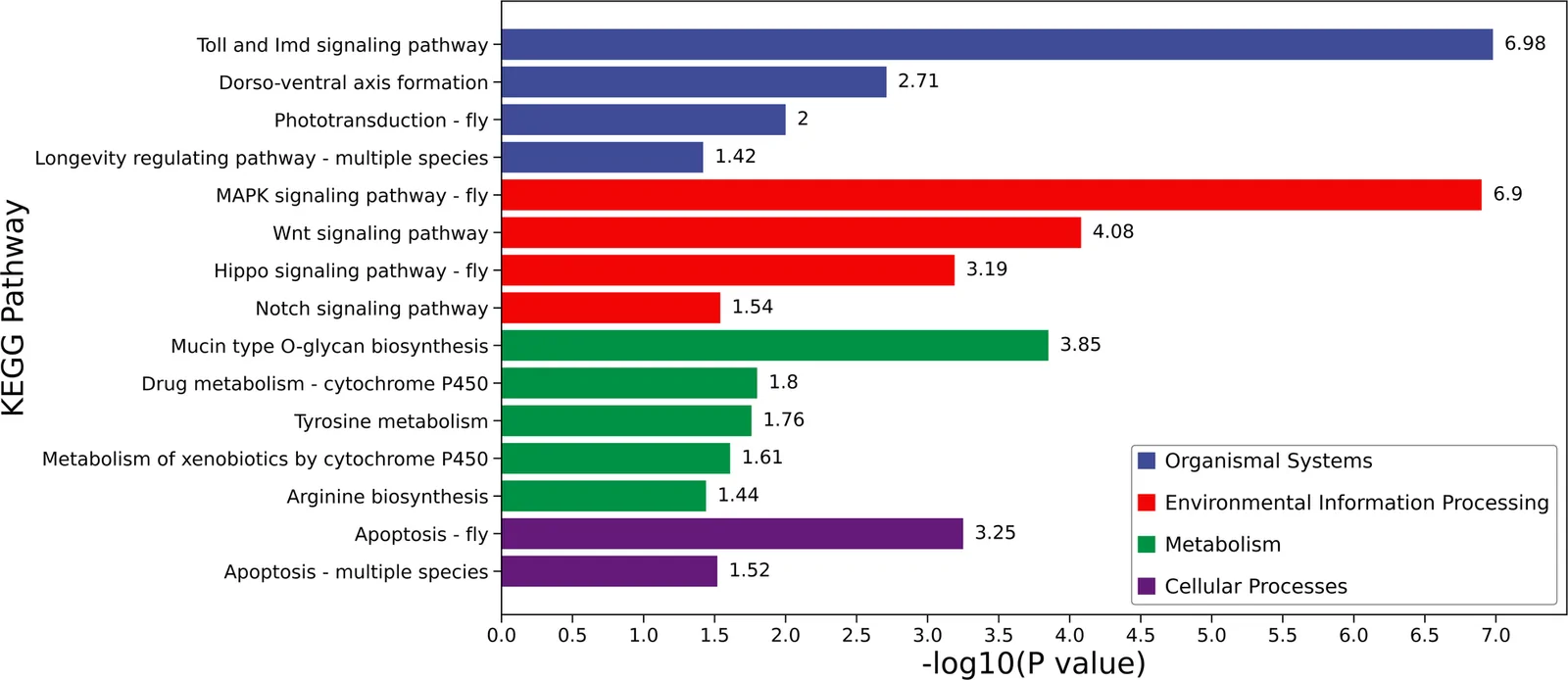
KEGG enrichment of key genes. The reference species is Silkworm, -log_10_(*P* value) is the *p* value in -log base 10

To further understand the biological functions of these high-weight gene families and identify the major metabolic pathways in which they participate, we analyzed the identified high-weight genes *D. melanogaster* using the Kyoto Encyclopedia of Genes and Genomes (https://www.kegg.jp/kegg/) [47]. The analysis of the results showed 97 genes were enriched in 8 pathways (*P* < 0.01) (Fig. 8; Table S5). Among these genes, we found 16 genes (MP1, modSP, and PGRP-SA et al.) are involved in the *Toll and Imd signaling* pathway [48], 23 genes (*bab2*,* ttk*, and Ras85D et al.) located in the *MAPK signaling pathway – fly* [49], 10 genes (*br*, Dronc, Ras85D, and Eip93F et al.) were located in the *Apoptosis – fly* [50], and 8 genes (*chinmo*,* ab*, and *aop et al.*) were located in the *Dorso-ventral axis formation* [51]. These enriched signaling pathways are closely associated with insect metamorphosis. Overall, the genes within these pathways reflect their necessity for immune defense [52], apoptosis [53], and development [54] during the transformation from larva to pupa and adult.

### Case study

**Fig. 9:**
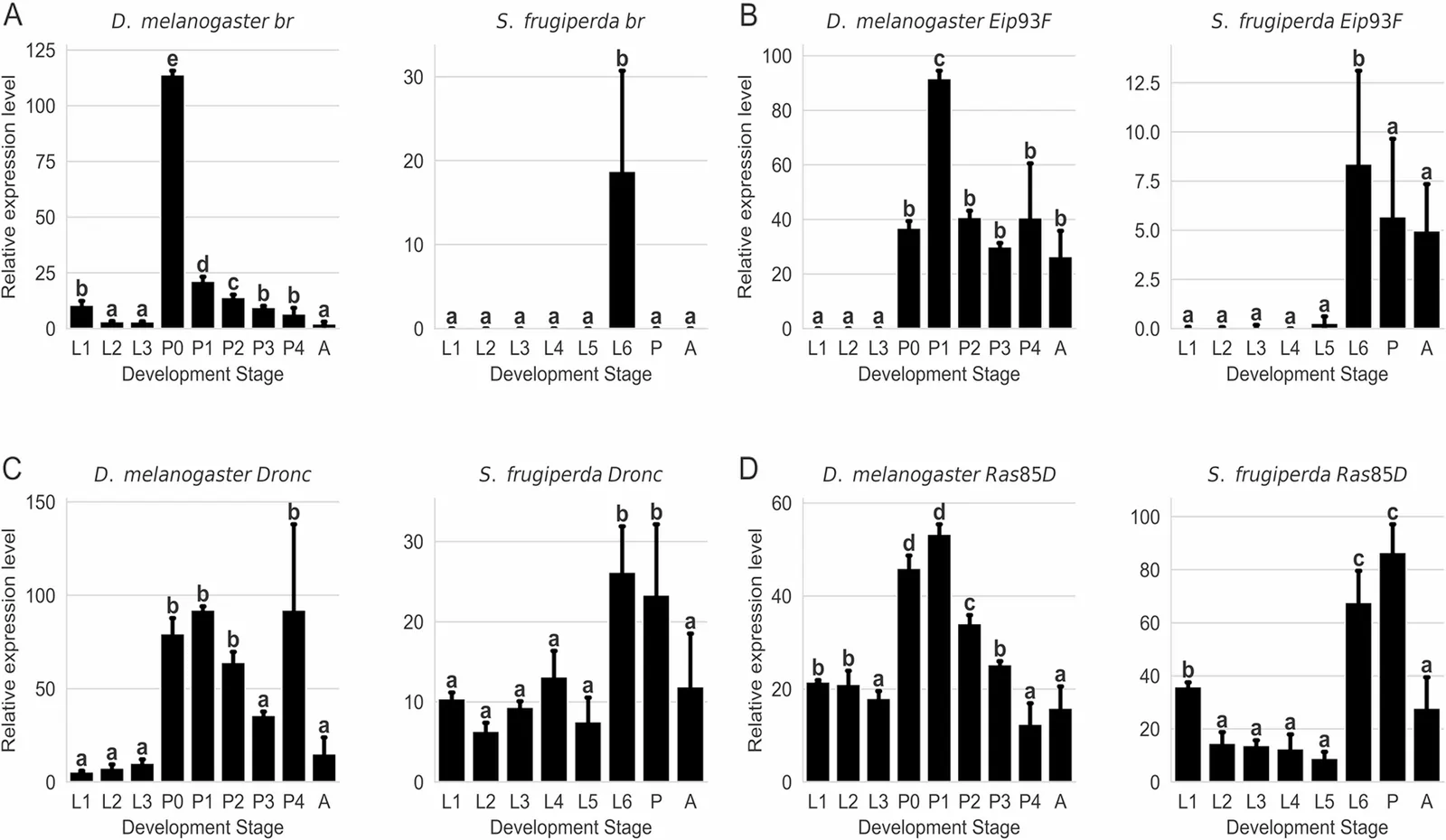
Relative expression levels of genes in the apoptosis pathway at different developmental stages in *D. melanogaster* and *S. frugiperda*. **A** *br* is upregulated during the prepupal stage. **B** Eip93F is expressed during the pupal stage. **C** *Dronc* is upregulated during the prepupal stage. **D** *Ras85D* is highly expressed during the pupal stage.The developmental stages were first instar larvae (L1), second instar larvae (L2), third instar larvae (L3), fourth instar larva (L4), fifth instar larva(L5), sixth instar larva(L6), and the pupal stage (P), prepupal stage (P0), first day of pupal stage (P1), second day of pupal stage (P2), third day of pupal stage (P3), fourth day of pupal stage (P4), and the first day of adult stage (A). Each bar is the average TPM of the stage, and the error line is the standard deviation (SD). Statistical tests were performed on log2(TPM + 1): one-way ANOVA (one-way ANOVA, log2TPM ~ Stage) was fitted to each gene and multiple comparisons (two-sided, α = 0.05) were performed with Tukey’s HSD. Different letters on the columns indicate significant differences between stages (same letter is not significant)

To further validate the ability of GNCM to identify key genes involved in insect pupation, we performed a differential analysis on the genes with higher and lower weights identified by GNCM. First, we analyzed the P0, P1, P2, P3, and P4 stages of *D. melanogaster* (Fig. S6). The results showed that the expression patterns of high-weight and low-weight genes differed significantly across all P0–P4 stages (Mann–Whitney U test, *p* < 0.05). Notably, at the P0(*p* = 6.62e–45), the expression levels of high-weight genes were significantly higher than those of low-weight genes, with the difference being most pronounced. At the P4(late pupal development), this difference remained statistically significant, although the p-value was significantly higher than in the previous stages. Second, we conducted case studies on different developmental stages of two holometabolous insect species: *D. melanogaster* and *S. frugiperda*. Holometabolous insects undergo extensive tissue removal and new structure formation during development, with programmed cell death (PCD) being a key event in this process [55–57]. Notably, the *br* (Broad-Complex), Eip93F (Ecdysone-induced protein 93 F), Dronc (Death regulator Nedd2-like caspase), and Ras85D (Ras oncogene at 85D) in the apoptosis pathway are highly expressed during the pupal stage of *D. melanogaster* and *S. frugiperda* (Fig. 9, Table S6), consistent with the physiological role of tissue apoptosis in clearing old tissues during metamorphosis. In summary, GNCM is capable of identifying key genes involved in the metamorphosis of insect pupae.

## Discussion

In recent years, with the rapid accumulation of genomic data and the continuous advancement of artificial intelligence technologies, DL has gradually emerged as a powerful tool for identifying key trait genes. DL can automatically learn latent features within large-scale sequences and achieve high-dimensional expression modeling by constructing complex feature spaces, thereby overcoming traditional methods’ reliance on manually set thresholds. This study designed a GNCM model based on SAM and BiGRU, using protein sequences as input to identify key genes involved in insect pupal metamorphosis. Compared to traditional machine learning methods, GNCM yields more precise results. When evaluated other algorithms such as BiGRU, BiLSTM, ANN, SVM, and XGBoost, GNCM demonstrated superior performance across all metrics (Tables 2, 3 and 4), attributed to its selective SAM. Compared to the feature extraction model based on the Sg sequence, the GNCM model built using CBOW demonstrates superior performance across all evaluation metrics (Figs. 5 and 6 ), likely because the features extracted by CBOW are smoother (Figs. 4D, E).

To validate the reliability of GNCM in identifying key genes, we performed temporal soft clustering analysis on stage-specific transcriptomes of *D. melanogaster* using Mfuzz software, focusing on gene clusters that peaked in expression during the pupal stage (Fig.S5). These gene clusters preferentially transcribed during the pupal stage, such as Clusters 6, Clusters 7, Clusters 8, Clusters 9, Clusters 10, Clusters 11, Clusters 12, Clusters 14, Clusters 15, Clusters 16, Clusters 18, and Clusters 19 (Fig.S5), these genes may play an important role in the metamorphosis process during the pupal stage. Although genes within the GNCM-identified families are not exclusively highly expressed during the pupal stage, this may result from the presence of non-essential genes within certain gene families. In addition, the genes identified by the GNCM as having higher weights exhibited higher expression levels during the pupal stage compared to those with lower weights (Fig.S6). There are 166 overlapping genes between the genes enriched in WGCNA during the pupal stage and GNCM, suggesting that GNCM is capable of identifying pupal-specific genes (Figs.S7,8). Regardless, the majority of genes identified by GNCM exhibit pupa-specific expression. These findings validate GNCM’s feasibility and accuracy in identifying insect pupal metamorphosis genes, laying a solid foundation for subsequent functional validation and mechanism studies.

Insects undergo a series of time-regulated morphological stages during development that lead to the final adult form. In holometabolous insects, the larva transitions through the pupal stage to the adult form, when the larval tissues are completely reorganized and adult structures such as wings gradually form [58]. Previous studies have revealed that *chinmo* is essential for maintaining the larval stage in *D. melanogaster*, and with *br* and Eip93F, it forms a triad of master genes governing insect metamorphosis [16]. The *chinmo* is strongly expressed during larval growth (Fig.S9A), and its removal leads to premature expression in *br* first-stage larvae, resulting in a shift from larval to pre-metamorphic function [59]. In holometabolous insects, *br* is specifically upregulated during the larval-to-pupal transition and is essential for pupal formation (Fig. 8A). Previous studies have shown that the *br* triggers pupation in holometabolous insects [60, 61], whereas in hemimetabolous insects, *br* is primarily involved in the development of wings and ovipositor [62]. The *Eip93F* acts as a marker for adult-specific differentiation during the pupal stage and plays a key role in programmed cell death, which is essential for mediating tissue remodeling [63–65]. Additionally, several genes in the Toll and Imd signaling pathway exhibit high expression during the pupal stage (Figs.S9B-D), such as PGRP-SA [66], MP1 [67], and modSP [68], we speculate that these genes may play a role in the immune system during the metamorphosis of insects. Some of the key genes identified by GNCM have been shown to be essential for insect metamorphosis (Table S8). These genes demonstrate the feasibility of GNCM in identifying key genes involved in the metamorphosis of insect pupae. Furthermore, some new insect pupal metamorphosis candidate genes have also been identified (Fig.S10). These candidate genes are significantly upregulated in the P0 stage (Figs.S10A, B) of *D. melanogaster*. These newly identified candidate genes require further validation and also provide a foundation for future research on insect metamorphosis.

This study has several limitations. First, due to the limited availability of genomic data from hemimetabolous insects for modeling, the unbalanced sampling may introduce some bias into the model results. Second, because of the limited transcriptomic data available, this study only examined gene expression differences between *D. melanogaster* and *S. frugiperda* at different developmental stages. As more insect genomes are sequenced and artificial intelligence technologies advance rapidly, these limitations will be effectively addressed. Although GNCM has identified many genes associated with insect metamorphosis, these may include both genuine biological signals related to metamorphosis and signals related to certain data structural patterns. In the future, it will be necessary to combine these findings with more rigorous phylogenetic control analyses, independent validation data, and functional experiments to further screen and validate these candidate genes, thereby more clearly distinguishing the contributions of “biological signals” from those of “dataset structural signals”.

## Conclusion

In this study, we constructed a gene network classification model (GNCM) based on BiGRU and SAM to identify key genes influencing insect metamorphosis. Comparative analysis across different dimensionality embeddings revealed that GNCM achieved optimal classification performance at 150 dimensions. When comparing sequence feature construction methods (BPE and HFkmer) with extraction methods (CBOW and Sg), the combination of CBOW and BPE yields the best results. Compared with algorithms such as SVM, XGBoost, ANN, BiGRU, and BiLSTM, GNCM demonstrated superior performance across all evaluation metrics. The high-weight gene families identified by GNCM predominantly exhibited clustering trends of high expression during the pupal stage, indicating its reliability in protein sequence classification and key gene identification tasks for hemimetabolous and holometabolous insects. This study not only identified multiple genes known to be associated with insect pupal metamorphosis but also discovered candidate genes that have not been studied yet and may play important roles in insect pupal metamorphosis, providing a reference for future research on insect metamorphic development.

## Supplementary Information

Supplementary Material 1.

Supplementary Material 2.

## Data Availability

All sequence information used for model construction and analysis was obtained from publicly accessible NCBI databases and is available in the figshare repository: [https://doi.org/10.6084/m9.figshare.30061399.v1]. Corresponding accession numbers are detailed in Supplementary Material 2 (Table S1 and S2). Other data supporting the findings of this study are available from the corresponding author upon reasonable request.

## Code availability

The source code for GNCM is available at the following link: https://github.com/liufangrong/GNCM.
