# Supplementary material for: A self-attention-based deep learning model for identifying key genes in insect pupal metamorphosis
Source: BMC Genomics. 2026 Jul 6;27:786. doi: 10.1186/s12864-026-13128-5 (PMC13617954; doi:10.1186/s12864-026-13128-5)
Supplement: Supplementary file 1 [file 12864_2026_13128_MOESM1_ESM.pdf]

Supplementary Material 1

A self-attention-based deep learning model for identifying key genes in insect pupal metamorphosis

Fangrong Li, Yong Cao, Songping Qian, Xingyu Tong, Junhui Liu, Yan Zhang, Jiaying Zhu, Jiawei Mao, Xingke Yang, Jiasheng Hao, Youjie Zhao

*Corresponding authors: bioala@swfu.edu.cn

Supplementary Figures1-10:

- Fig S1. The process of HFkmer tokenization.
- Fig S2. The Skip-Gram word embedding algorithm for Word2vec.
- Fig S3. Process of obtaining attention weights.
- Fig S4. Distribution of the phylogeny of 293 insects.
- Fig S5. Dynamics of gene expression along *D. melanogaster* life cycle.
- Fig S6. Comparison of expression distributions between high-weight and low-weight gene groups across P0–P4 stages.
- Fig S7. WGCNA clustering results for different developmental stages.
- Fig S8. Overlapping genes enriched in GNCM and WGCNA during the pupal development stage.
- Fig S9. Relative expression of key genes at different developmental stages in D. melanogaster.
- Fig S10. The new genes have been identified by GNCM.

Supplementary Table S7-S8:

Table S7. A comparison of the performance of attention-free models on the test set under different sequence segmentation and word embedding schemes.

Table S8. The key genes identified by GNCM have been shown to be essential for insect metamorphosis.

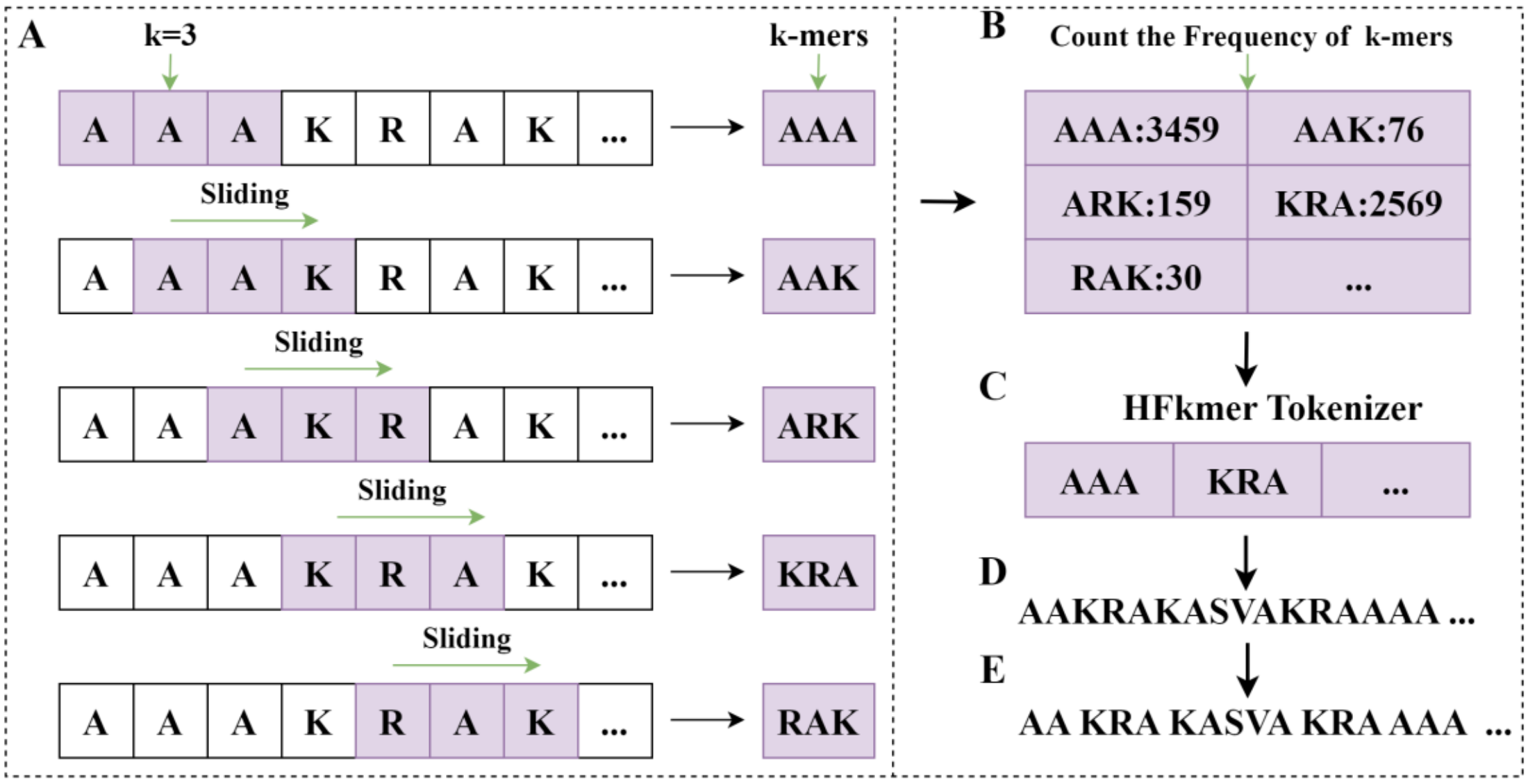

Fig S1. The process of HFkmer tokenization. **(A)** The sliding window disambiguation. **(B)** The frequency of each k-mers occurrence. **(C)** The selection of high-frequency k-mers as a participle marker, called HFkmer. **(D)** The original genomic protein sequence. **(E)** The result of HFkmer participle.

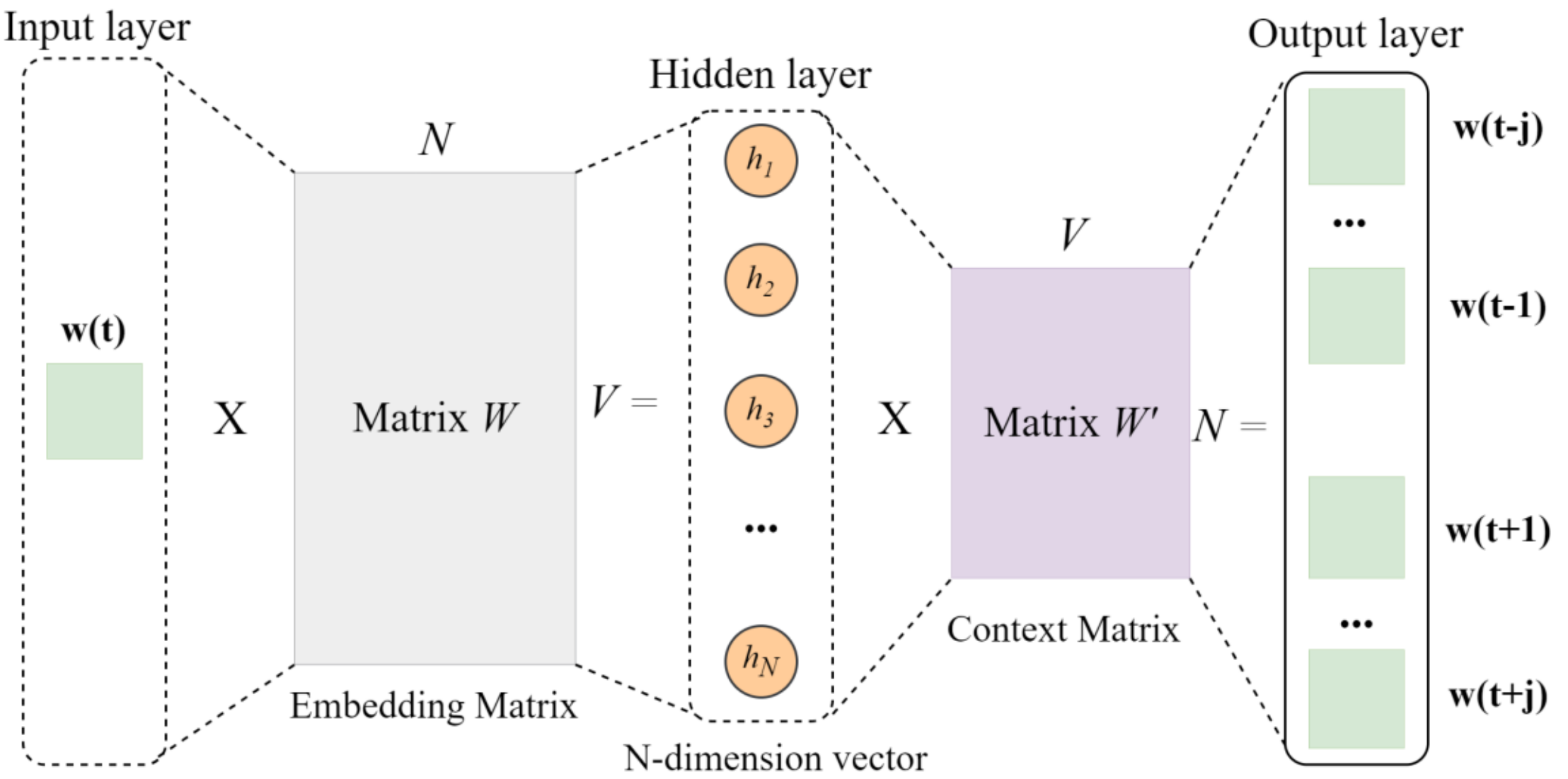

Fig S2. The Skip-Gram word embedding algorithm for Word2vec. It predicts the center word by context words. The context window of Skip-Gram word embedding model in this study is set to 20, the embedding dimension is 150, and the number of training rounds is 5.

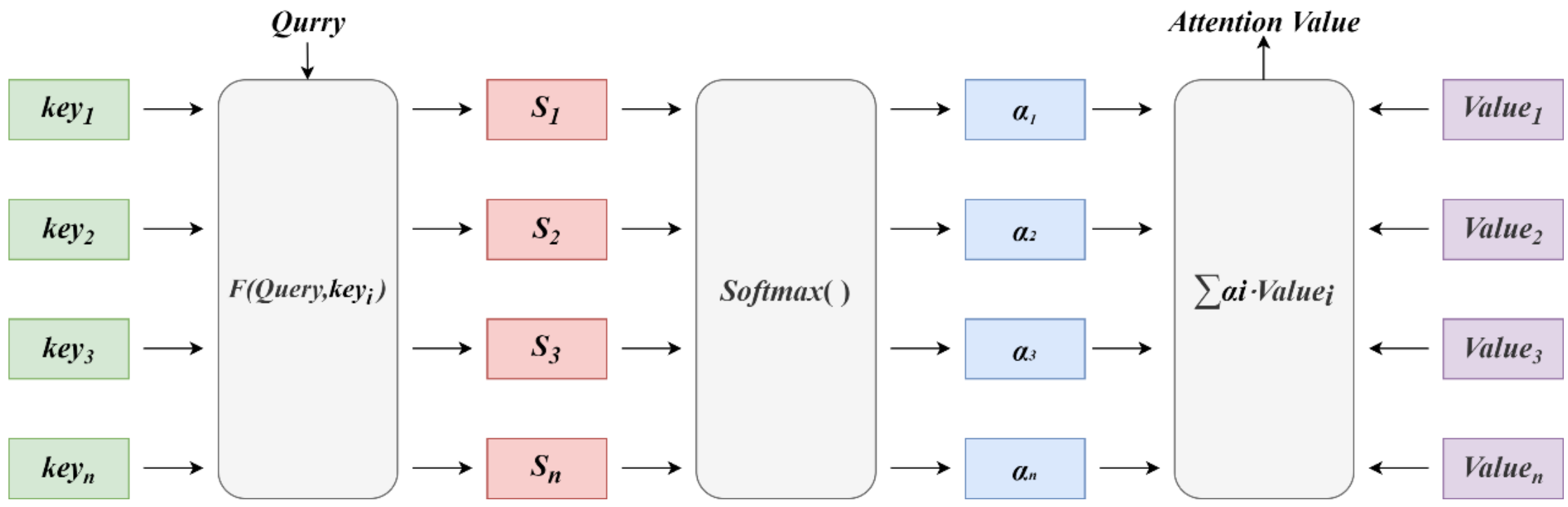

Fig S3. Process of obtaining attention weights. If the model can be given its correct classification label, the output of the attention layer during the calculation can be used as the importance distribution of the genes in the classification process.

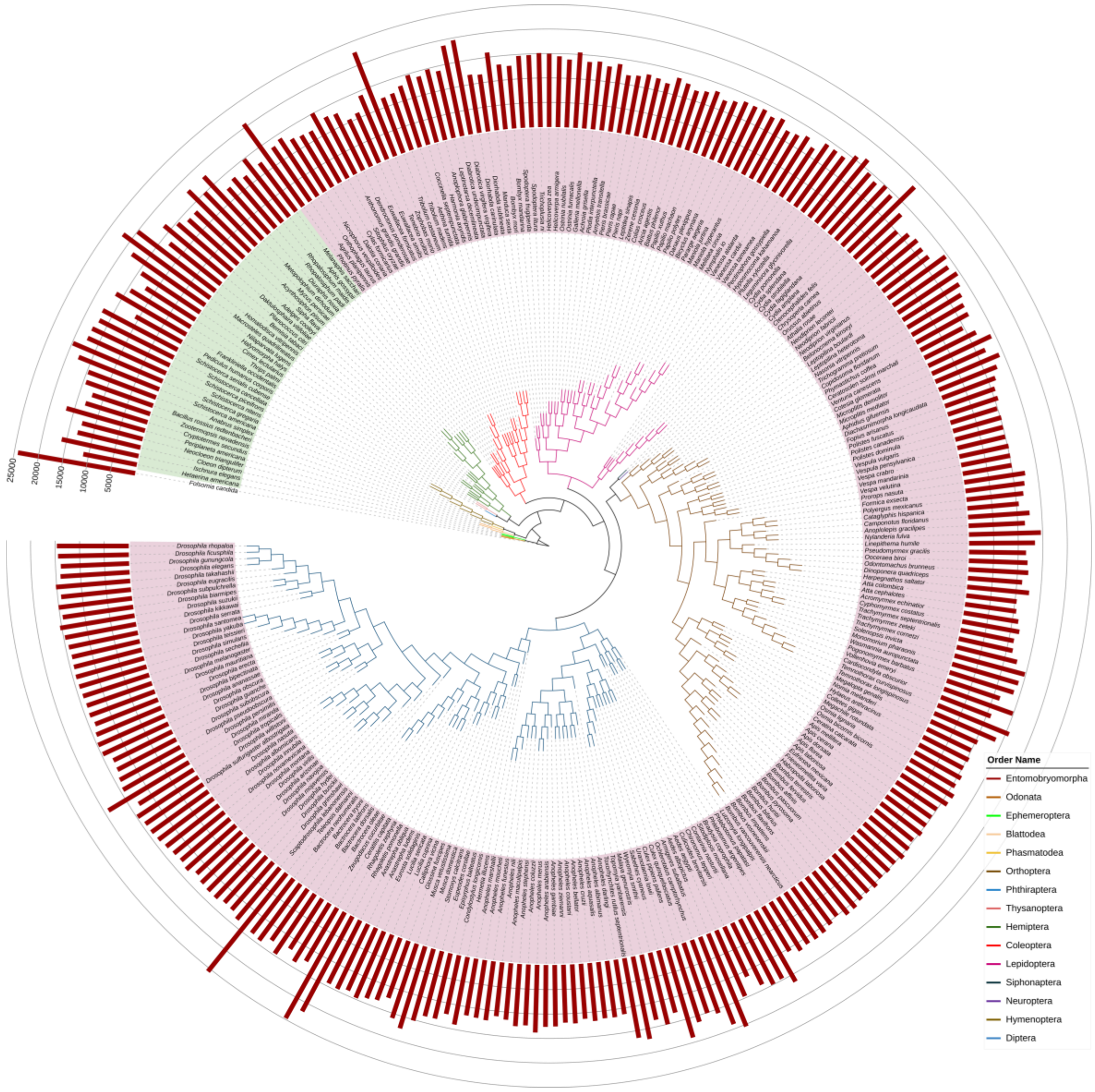

Fig S4. Distribution of the phylogeny of 293 insects. 293 insects are distributed across 81 families in 14 orders. The bar chart shows the distribution of the number of longest transcripts for each insect. *Folsomia candida* belongs to the outgroup species. Phylogenetic trees were constructed online through NCBI (https://www.ncbi.nlm.nih.gov/taxonomy/) and iTOL(https://itol.embl.de/).

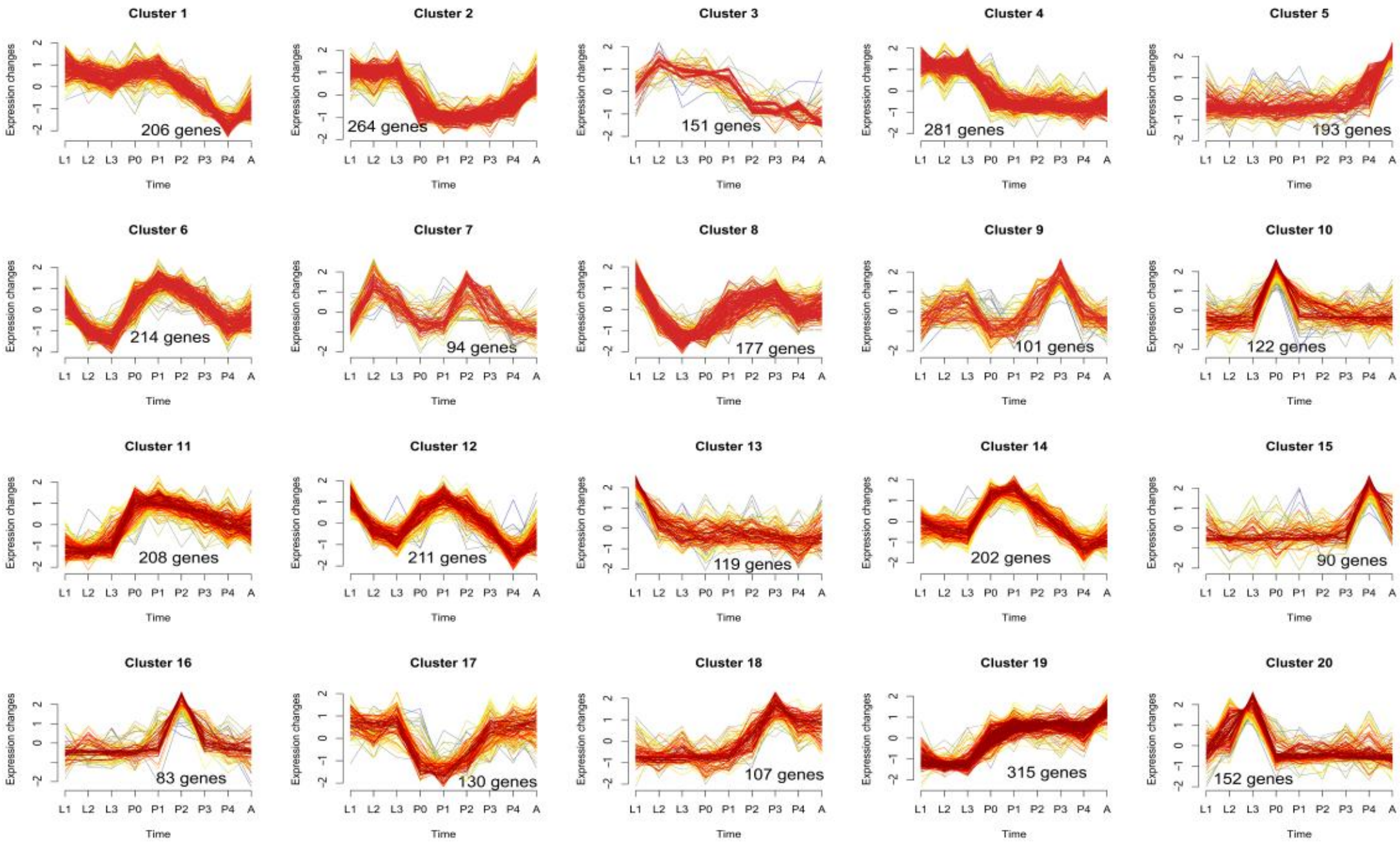

Fig S5. Dynamics of gene expression along *D. melanogaster* life cycle. Relative expression changes along RNA-seq samples, corresponding to different life cycle stages obtained with Mfuzz R package (L1: first instar larvae, L2: second instar larvae, L3: third instar larvae, P0: prepupae, P1: first day of pupation, P2: second day of pupation, P3: third day of pupation, P4: fourth day of pupation, A: first day of adulthood). Numbers in plot show the number of genes assigned to each cluster.

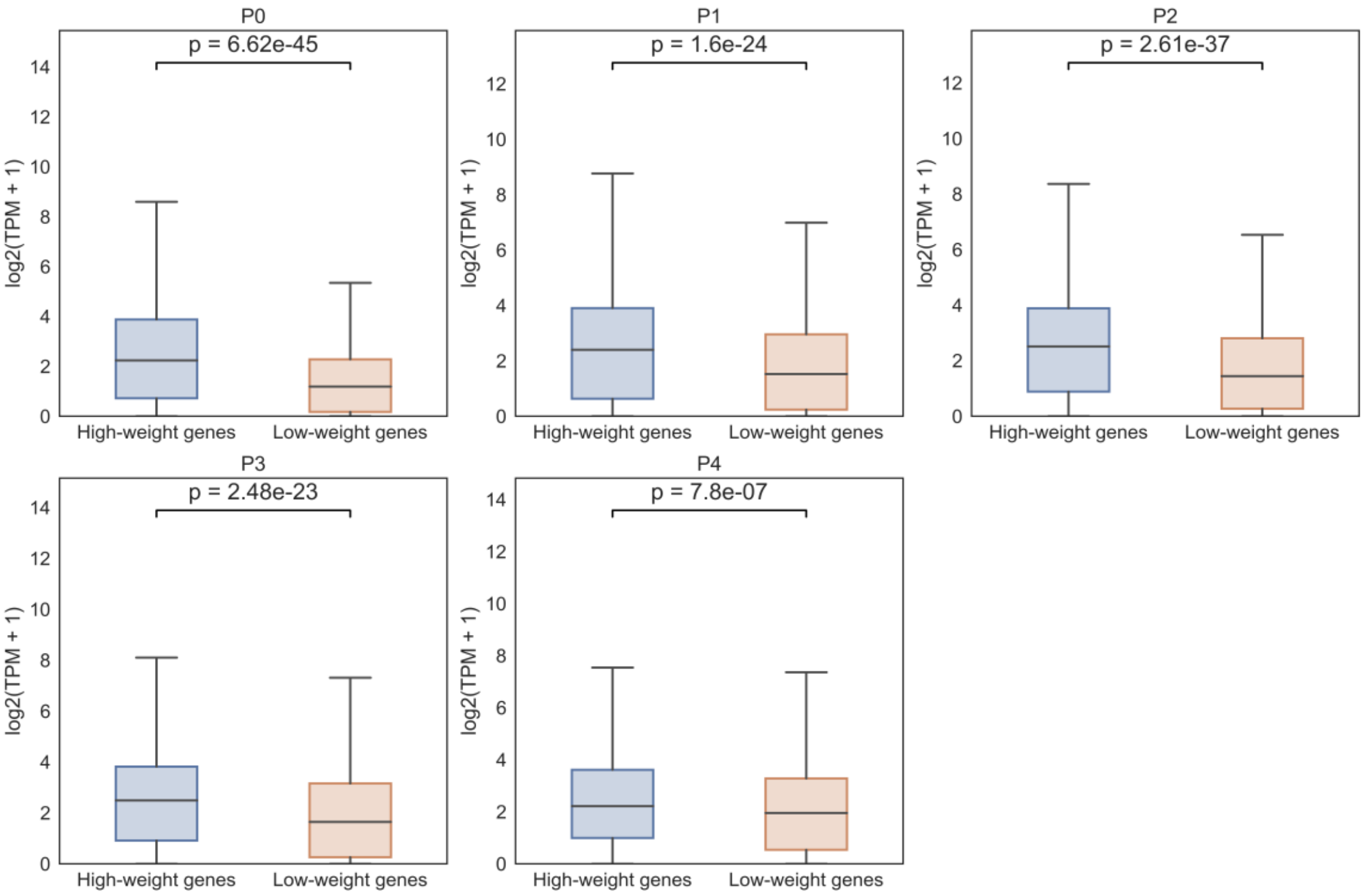

Fig S6. Comparison of expression distributions between high-weight and low-weight gene groups across P0–P4 stages. The developmental stages were prepupal stage (P0), first day of pupal stage (P1), second day of pupal stage (P2), third day of pupal stage (P3), and fourth day of pupal stage (P4). Boxplots show the expression differences between high-weight genes and low-weight genes across P0, P1, P2,

P3, and P4 stages. Expression values were represented as TPM and visualized after log2(TPM+1) transformation of the mean expression across replicates for each stage. The central line indicates the median, the box bounds represent the interquartile range, and the whiskers indicate the spread of the data. Statistical significance between the two groups at each stage was evaluated using the Mann–Whitney U test, and the corresponding p-values are shown above each boxplot.

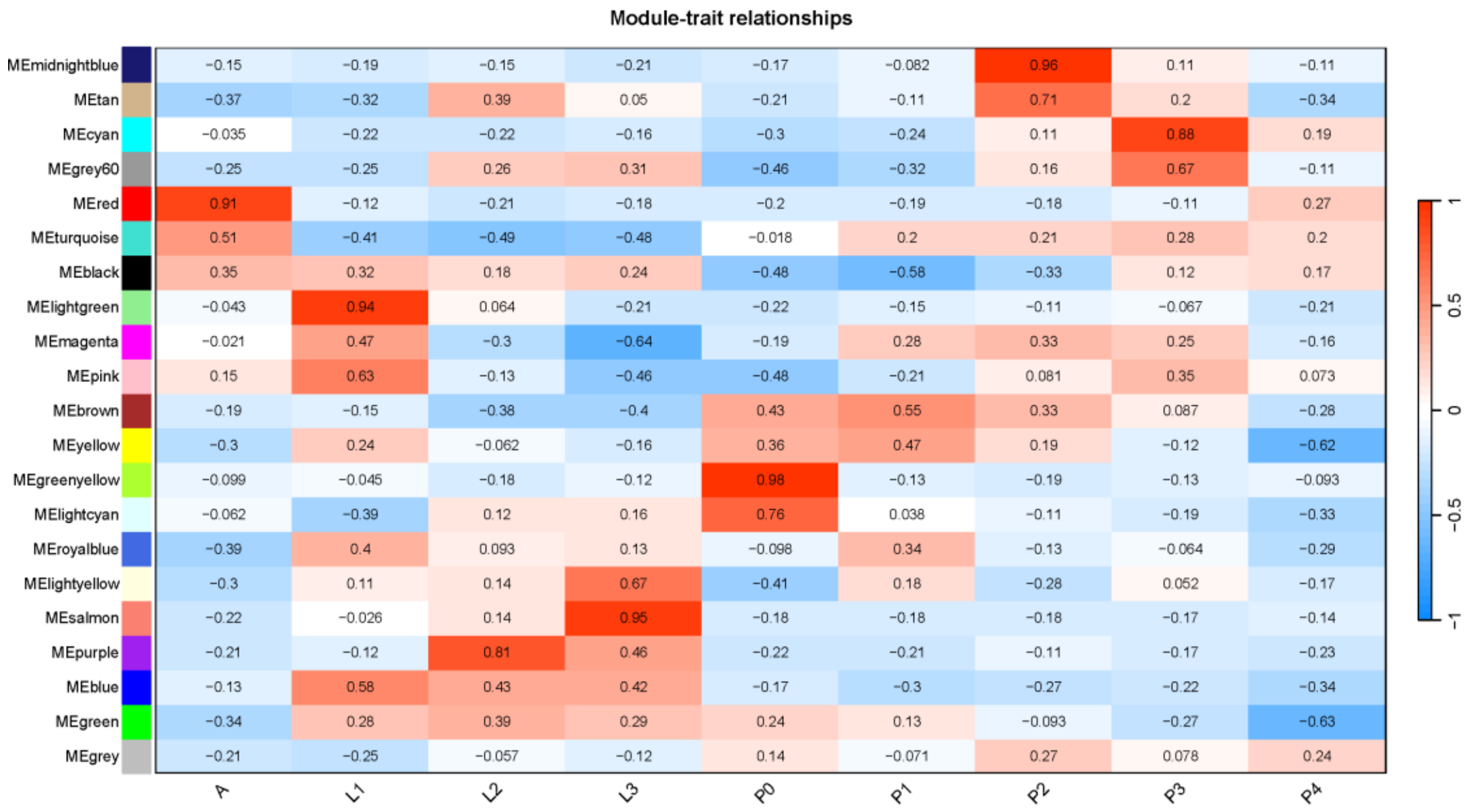

Fig S7. WGCNA clustering results for different developmental stages. The networks between genes and modules using *D. melanogaster* as a model organism while ensuring that $R^2 > 0.85$ for optimal network connectivity.

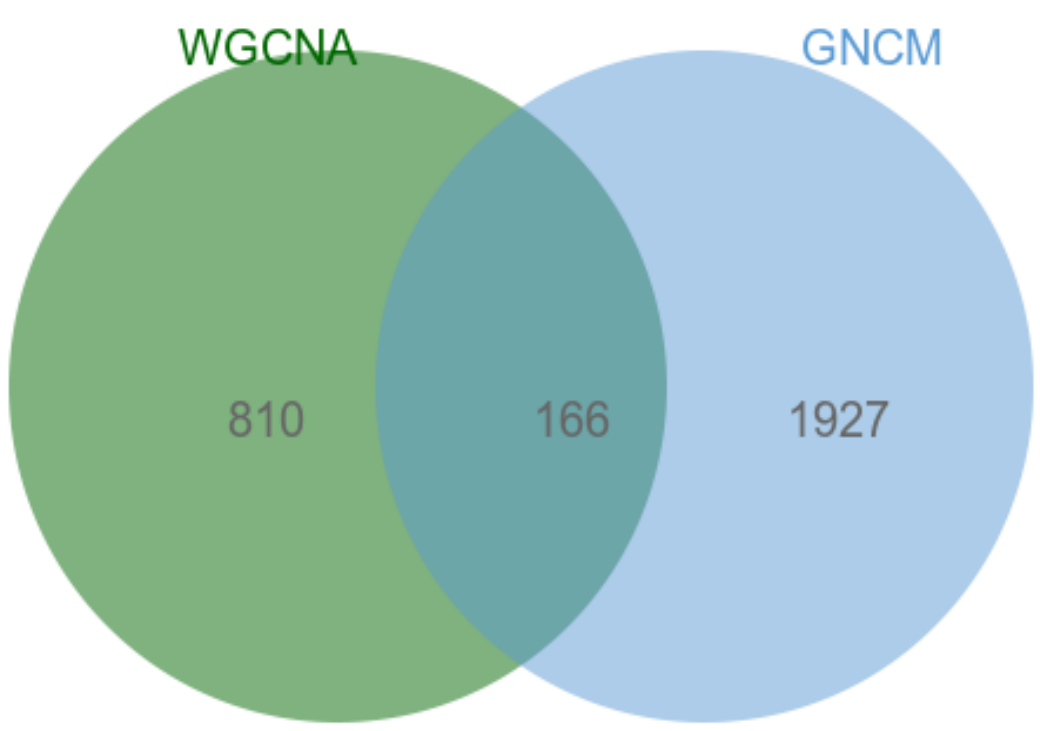

Fig S8. Overlapping genes enriched in GNCM and WGCNA during the pupal development stage. WGCNA was used to identify genes enriched in the P0, P1, P2, P3, and P4 stages of Drosophila melanogaster, while GNCM identified high-weight candidate genes with well-defined gene name annotations.

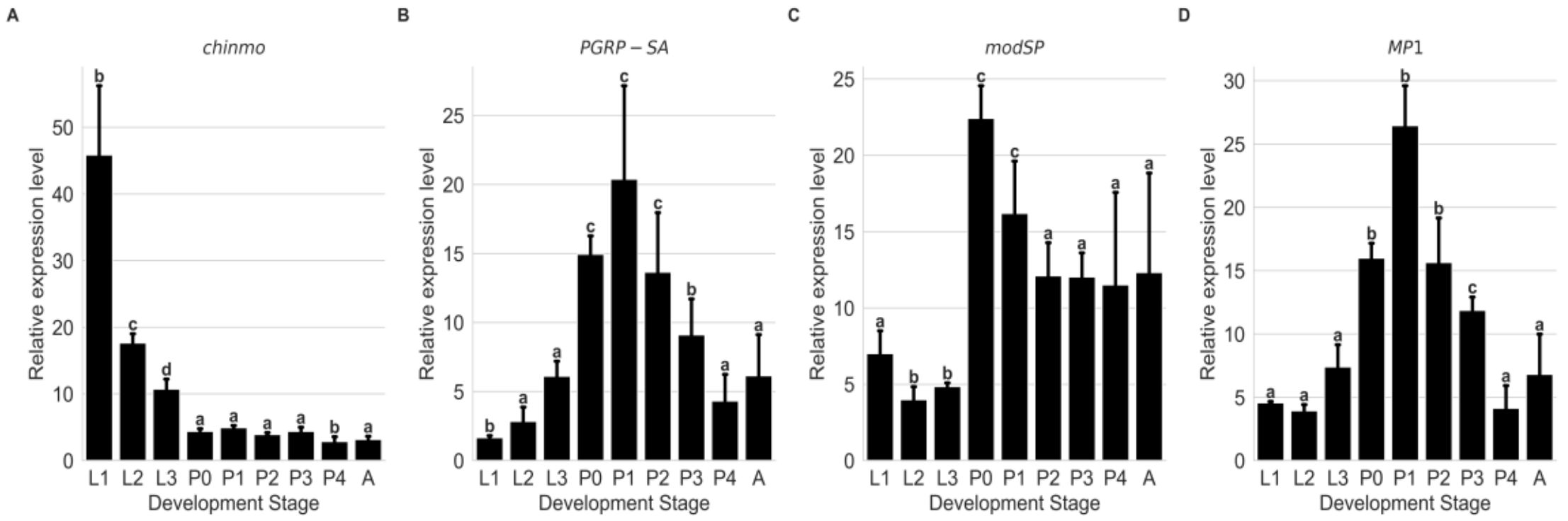

Fig S9. Relative expression of key genes at different developmental stages in D. melanogaster. The developmental stages were first instar larvae (L1), second instar larvae (L2), third instar larvae (L3), prepupal stage (P0), first day of pupal stage (P1), second day of pupal stage (P2), third day of pupal stage (P3), fourth day of pupal stage (P4), and the first day of adult stage (A). Each bar is the average TPM of the stage, and the error line is the standard deviation (SD). Statistical tests were performed on log2(TPM+1): one-way ANOVA (one-way ANOVA, log2TPM ~ Stage) was fitted to each gene and multiple comparisons (two-sided, $\alpha = 0.05$) were performed with Tukey's HSD. Different letters on the columns indicate significant differences between stages (same letter is not significant).

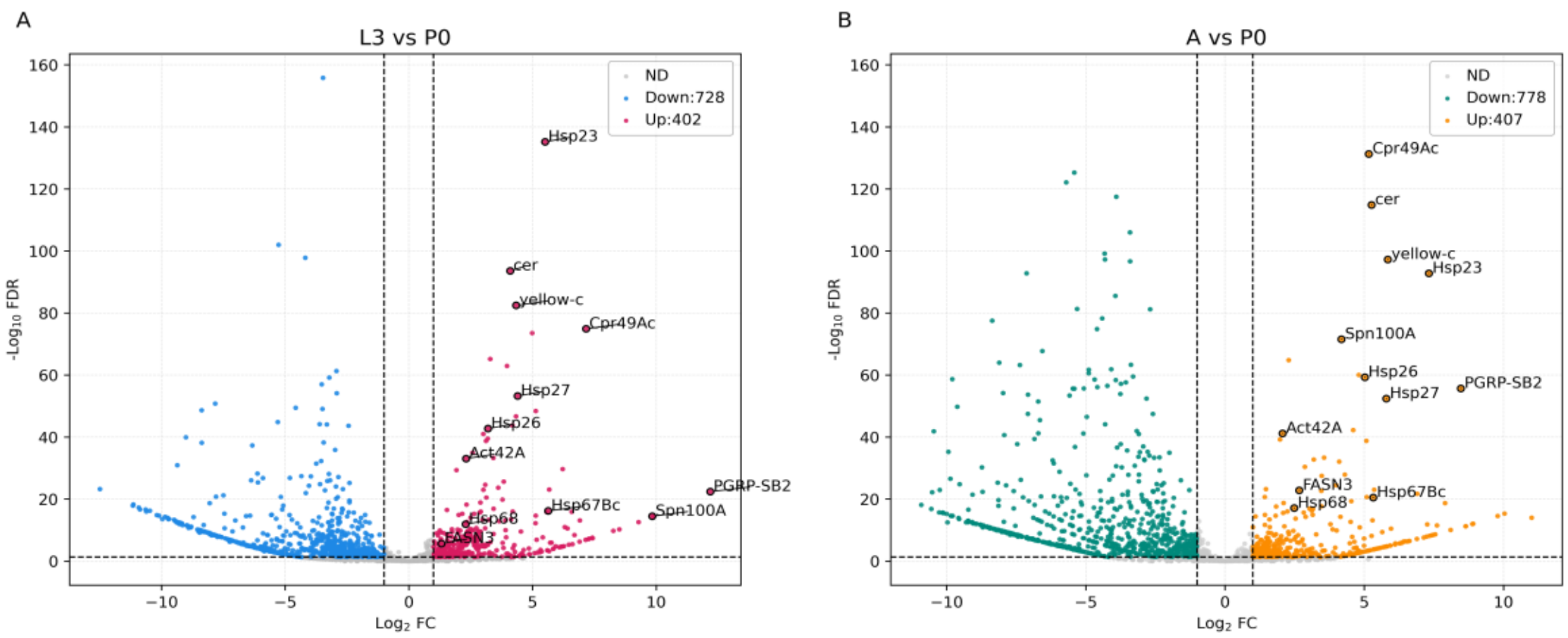

Fig S10. The new genes have been identified by GNCM. (A) Differential expression analysis of genes in D. melanogaster L3 vs. P0. **(B)** Differential expression analysis of genes in D. melanogaster A vs. P0. The analytical method was DESeq2, and the multiplicity of differences was 2. The threshold $|log_2FC| \geq 1$ and FDR < 0.01, a total of 1,130 differential genes were identified in L3 vs P0 (402 up-regulated genes, 728 down-regulated genes), and a total of 1,185 genes were identified in A vs P0 (407 up-regulated genes, 778 down-regulated genes).

Table S7. A comparison of the performance of attention-free models on the test set under different sequence segmentation and word embedding schemes.

Algorithm	Accuracy	F1	Precision	Recall	CrossEntropyLoss
BPE+CB+BiGRU	**0.7530**	**0.6998**	**0.7845**	**0.6919**	**0.5443**
BPE+CB+BiLSTM	0.7247	0.6720	0.7305	0.6670	0.5761
BPE+SG+BiLSTM	0.6412	0.6247	0.6605	0.6359	0.6517
BPE+SG+BiGRU	0.6412	0.6247	0.6605	0.6359	0.6489
HFkmer+SG+BiGRU	0.6412	0.6247	0.6605	0.6359	0.6464
HFkmer+SG+BiLSTM	0.6412	0.6247	0.6605	0.6359	0.6487
HFkmer+CB+BiGRU	0.6412	0.6247	0.6605	0.6359	0.6466
HFkmer+CB+BiLSTM	0.6412	0.6247	0.6605	0.6359	0.6506

Table S8. The key genes identified by GNCM have been shown to be essential for insect metamorphosis.

Gene Symbol	Description	Author
br	broad-complex	Erezyilmaz et al. [1]
chinmo	Chronologically inappropriate morphogenesis	Truman et al. [2]
Eip93F	Ecdysone-induced protein 93F	Wang et al. [3]
Eip75B	Ecdysone-induced protein 75B	Huet et al.[4]
Eip78C	Ecdysone-induced protein 78C	Stone et al. [5]
Eip74EF	Ecdysone-induced protein 74EF	Zhang et al. [6]
Eo	Ecdysone oxidase	Li et al. [7]
Pcp	Pupal cuticle protein	Li et al. [8]
Edg78E	Ecdysone-dependent gene 78E	Kawasaki et al. [9]
Jhe	Juvenile hormone esterase	Chanchay et al. [10]
Sox14	Sox box protein 14	Luo et al. [11]
Dronc	Death regulator Nedd2-like caspase	Dorstyn et al. [12]
Fbp1	Fat body protein 1	Yu et al. [13]
Aldh	Aldehyde dehydrogenase	Rivera-Perez et al. [14]
Mmp2	Matrix metalloproteinase 2	Jia et al. [15]
Mmp1	Matrix metalloproteinase 1	Jia et al. [15]
Drice	Death related ICE-like caspase	Fraser et al. [16]
Bab2	bric a brac 2	Duan et al. [17]
HmgD	High mobility group protein D	Chen et al. [18]
Strica	Ser/Thr-rich caspase	Doumanis et al. [19]
Obst-E	obstructor-E	Tajiri et al. [20]
USP	ultraspiracle	Yu et al. [21]
Hr78	Hormone-receptor-like in 78	Huang et al. [22]
Hr83	Hormone receptor 83	Xu et al. [23]
Tai	Taiman	Xu et al. [24]
Blimp-1	Blimp-1	Wu et al. [25]
Sgs5	Salivary gland secretion 5	Lage et al.[26]
Sgs5bis	Sgs5bis	Lage et al.[26]
shep	alan shepard	Chen et al. [27]

References

1. Erezyilmaz DF, Riddiford LM, Truman JW. The pupal specifier broad directs progressive morphogenesis in a direct-developing insect. Proceedings of the National Academy of Sciences. 2006;103: 6925–6930. doi:10.1073/pnas.0509983103
2. Truman JW, Riddiford LM. Chinmo is the larval member of the molecular trinity that directs Drosophila metamorphosis. Proceedings of the National Academy of Sciences. 2022;119: e2201071119. doi:10.1073/pnas.2201071119

3. Wang X, Ding Y, Lu X, Geng D, Li S, Raikhel AS, et al. The ecdysone-induced protein 93 is a key factor regulating gonadotrophic cycles in the adult female mosquito Aedes aegypti. Proceedings of the National Academy of Sciences. 2021;118: e2021910118. doi:10.1073/pnas.2021910118
4. Huet F, Ruiz C, Richards G. Puffs and PCR: the in vivo dynamics of early gene expression during ecdysone responses in Drosophila. Development. 1993;118: 613–627. doi:10.1242/dev.118.2.613
5. Stone BL, Thummel CS. The Drosophila 78C early late puff contains E78, an ecdysone-inducible gene that encodes a novel member of the nuclear hormone receptor superfamily. Cell. 1993;75: 307–320. doi:10.1016/0092-8674(93)80072-m
6. Zhang S, Wu S, Yao R, Wei X, Ohlstein B, Guo Z. Eclosion muscles secrete ecdysteroids to initiate asymmetric intestinal stem cell division in Drosophila. Developmental Cell. 2024;59: 125-140.e12. doi:10.1016/j.devcel.2023.11.016
7. Li Z, You L, Zeng B, Ling L, Xu J, Chen X, et al. Ectopic expression of ecdysone oxidase impairs tissue degeneration in Bombyx mori. Proceedings of the Royal Society B: Biological Sciences. 2015;282: 20150513. doi:10.1098/rspb.2015.0513
8. Li A, Denlinger DL. Pupal cuticle protein is abundant during early adult diapause in the mosquito Culex pipiens. J Med Entomol. 2009;46: 1382–1386. doi:10.1603/033.046.0618
9. Kawasaki H, Hirose S, Ueda H. BetaFTZ-F1 dependent and independent activation of Edg78E, a pupal cuticle gene, during the early metamorphic period in Drosophila melanogaster. Dev Growth Differ. 2002;44: 419–425. doi:10.1046/j.1440-169x.2002.00655.x
10. Chanchay P, Vongsangnak W, Thancharoen A, Sriboonlert A. Reconstruction of insect hormone pathways in an aquatic firefly, Sclerotia aquatilis (Coleoptera: Lampyridae), using RNA-seq. PeerJ. 2019;7: e7428. doi:10.7717/peerj.7428
11. Luo G-H, Chen X-E, Jiao Y-Y, Zhu G-H, Zhang R, Dhandapani RK, et al. SoxC is Required for Ecdysteroid Induction of Neuropeptide Genes During Insect Eclosion. Front Genet. 2022;13: 942884. doi:10.3389/fgene.2022.942884
12. Dorstyn L, Colussi PA, Quinn LM, Richardson H, Kumar S. DRONC, an ecdysone-

inducible Drosophila caspase. Proceedings of the National Academy of Sciences. 1999;96: 4307–4312. doi:10.1073/pnas.96.8.4307

13. Yu Y-C, Lu H, Chiang Y-C, Tsai C-L, Zuo Y-H, Chen M-E. Molecular Characteristics of Fat Body Protein 1 in the Oriental Fruit Fly, Bactrocera dorsalis. Insects. 2021;12: 319. doi:10.3390/insects12040319
14. Rivera-Perez C, Nouzova M, Clifton MarkE, Garcia EM, LeBlanc E, Noriega FG. Aldehyde Dehydrogenase 3 Converts Farnesal into Farnesoic Acid in the Corpora Allata of Mosquitoes. Insect Biochem Mol Biol. 2013;43: 675–682. doi:10.1016/j.ibmb.2013.04.002
15. Jia Q, Li S. Mmp-induced fat body cell dissociation promotes pupal development and moderately averts pupal diapause by activating lipid metabolism. Proceedings of the National Academy of Sciences. 2023;120: e2215214120. doi:10.1073/pnas.2215214120
16. Fraser AG, McCarthy NJ, Evan GI. drICE is an essential caspase required for apoptotic activity in Drosophila cells. The EMBO Journal. 1997;16: 6192–6199. doi:10.1093/emboj/16.20.6192
17. Duan J, Zhao Y, Li H, Habernig L, Gordon MD, Miao X, et al. Bab2 Functions as an Ecdysone-Responsive Transcriptional Repressor during Drosophila Development. Cell Rep. 2020;32: 107972. doi:10.1016/j.celrep.2020.107972
18. Chen J, Wang H, Wang Y-F. Overexpression of HmgD causes the failure of pupariation in Drosophila by affecting ecdysone receptor pathway. Arch Insect Biochem Physiol. 2008;68: 123–133. doi:10.1002/arch.20237
19. Doumanis J, Quinn L, Richardson H, Kumar S. STRICA, a novel Drosophila melanogaster caspase with an unusual serine/threonine-rich prodomain, interacts with DIAP1 and DIAP2. Cell Death Differ. 2001;8: 387–394. doi:10.1038/sj.cdd.4400864
20. Tajiri R, Ogawa N, Fujiwara H, Kojima T. Mechanical Control of Whole Body Shape by a Single Cuticular Protein Obstructor-E in Drosophila melanogaster. PLoS Genet. 2017;13: e1006548. doi:10.1371/journal.pgen.1006548
21. Yu J, Song H, Wang Y, Liu Z, Wang H, Xu B. 20-hydroxyecdysone Upregulates Ecdysone Receptor (ECR) Gene to Promote Pupation in the Honeybee, Apis mellifera

Ligustica. Integr Comp Biol. 2023;63: 288–303. doi:10.1093/icb/icad077

22. Huang Y, Gong X, Yu L, Yin Y, Huang Y, Jiang M, et al. AaHR78 mediates the effects of 20E on growth and reproduction in Aedes aegypti. J Insect Physiol. 2025;162: 104790. doi:10.1016/j.jinsphys.2025.104790
23. Xu X, Pu S, Jiang M, Hu X, Wang Q, Yu J, et al. Knockout of nuclear receptor HR38 gene impairs pupal–adult development in silkworm Bombyx mori. Insect Molecular Biology. 2024;33: 29–40. doi:10.1111/imb.12876
24. Xu Q-Y, Du J-L, Mu L-L, Guo W-C, Li G-Q. Importance of Taiman in Larval-Pupal Transition in Leptinotarsa decemlineata. Front Physiol. 2019;10: 724. doi:10.3389/fphys.2019.00724
25. Wu S-Y, Tong X-L, Li C-L, Ding X, Zhang Z-L, Fang C-Y, et al. BmBlimp-1 gene encoding a C2H2 zinc finger protein is required for wing development in the silkworm Bombyx mori. Int J Biol Sci. 2019;15: 2664–2675. doi:10.7150/ijbs.34743
26. Da Lage J-L, Thomas GWC, Bonneau M, Courtier-Orgogozo V. Evolution of salivary glue genes in Drosophila species. BMC Evolutionary Biology. 2019;19: 36. doi:10.1186/s12862-019-1364-9
27. Chen D, Gu T, Pham TN, Zachary MJ, Hewes RS. Regulatory Mechanisms of Metamorphic Neuronal Remodeling Revealed Through a Genome-Wide Modifier Screen in Drosophila melanogaster. Genetics. 2017;206: 1429–1443. doi:10.1534/genetics.117.200378
